# Correction: Maternal smoking during pregnancy and offspring body composition in adulthood: results from two birth cohort studies

**DOI:** 10.1136/bmjopen-2018-023852corr1

**Published:** 2019-06-25

da Silva Magalhães EI, Peixoto Lima N, Baptista Menezes AM, *et al*. Maternal smoking during pregnancy and offspring body composition in adulthood: results from two birth cohort studies *BMJ Open* 2019;9:e023852. doi: 10.1136/bmjopen-2018-023852.

This article was previously published with an error.

The author name ‘MariaCecília Formoso Assunção’ should be ‘Maria Cecília Formoso Assunção’.

Author names in the to cite section have been changed to Magalhães EIS, Lima NP, Menezes AMB.

Table 5 footnote, ‘The significance of the values in bold can be verified by the 95% confidence intervals.' has been removed.

