# Supplementary material for: Maternal smoking during pregnancy and offspring body composition in adulthood: Results from two birth cohort studies
Source: BMJ Open. 2019 Jun 12;9(6):e023852. doi: 10.1136/bmjopen-2018-023852 (PMC6575638; doi:10.1136/bmjopen-2018-023852)
Supplement: Supplementary data [file bmjopen-2018-023852supp001.pdf]

Supplementary table 1. Description of follow-ups in the 1982 and 1993 cohorts, Pelotas - RS, Brazil.

1982 Cohort			1993 Cohort		
**Follow-up**	**Sample evaluated**	**Follow-up rate (%)**	**Follow-up**	**Sample evaluated**	**Follow-up rate (%)**
1982 (perinatal study)	All newborns	-	1993 (perinatal study)	All newborns	-
1983 (at 1st year)	All subjects born between January and April (1/3 of the cohort)	79,3	1993 (at 1st month)	Systematic sampling of 13% of cohort members	99,1
1984 (at 2 years)	All subjects	87,2	1993 (at 3 months)	Systematic sampling of 13% of cohort members	98,3
1986 (at 4 years)	All subjects	84,1	1993 (at 6 months)	All subjects with low birth weight and 20% of remnants	96,8
1997 (at 15 years)	All subjects residing in 27% of the city's census tracts	71,8	1994 (at 1st year)	All subjects with low birth weight and 20% of remnants	93,4
2000 (at 18 years)	All men	78,9	1997 (at 4 years)	All subjects with low birth weight and 20% of remnants	87,2
2001 (at 19 years)	All subjects residing in 27% of the city's census tracts	69,0	2004 (at 11 years)	All subjects	87,5
2004-2005 (at 23 years)	All subjects	77,4	2008 (at 15 years)	All subjects	85,7
2012-2013 (at 30 years)	All subjects	68,1	2011 (at 18 years)	All subjects	81,4
			2015-2016 (at 23 years)	All subjects	76,3

Supplementary table 2. Follow-up rate at 30 years (1982 cohort) and at 22 years of age (1993 cohort) according to baseline characteristics of the cohort.

Variable	1982 Cohort		1993 Cohort	
	Original cohort (n)	Followed at 30 years (%)	Original cohort (n)	Followed at 22 years (%)
**Sex**				
Male	3,037	65.2	2,603	72.6
Female	2,876	71.1	2,645	79.9
**Birthweight (g)**				
< 2500	534	72.1	510	79.2
2500 to 2999	1,393	69.1	1,409	75.7
3000 to 3499	2,220	66.0	2,030	75.9
≥ 3500	1,762	68.6	1,283	76.0
**Gestational age (weeks)**				
< 37	294	74.5	589	74.1
≥ 37	4,380	68.1	4,582	76.5
**Family income at birth (minimum wages)**				
< 1	1,288	66.1	967	75.1
1.1 to 3	2,789	70.4	2,260	74.8
3.1 to 6	1,091	69.3	1,204	79.6
6.1 to 10	382	61.3	433	75.7
≥ 10	335	60.3	385	74.6

Supplementary table 3. Maternal smoking during pregnancy according to baseline socioeconomic characteristics of the 1982 and 1993 cohorts.

	Duration of maternal smoking			
	**Non-smokers N (%)**	**Smoked in part of pregnancy N (%)**	**Smoked in whole Pregnancy N (%)**	**P-value**
**1982 Cohort**				
**Familiar income (tertile)**				< 0.0001
1st	682 (58.9)	90 (7.8)	385 (33.3)	
2nd	837 (63.9)	98 (7.5)	374 (28.6)	
3rd	880 (71.6)	93 (7.6)	255 (20.8)	
**Maternal schooling (years)**				< 0.0001
0 to 4	1,734 (62.4)	214 (7.7)	829 (29.9)	
5 to 8	273 (67.6)	33 (8.2)	98 (24.2)	
≥ 9	391 (76.5)	34 (6.7)	86 (16.8)	
**Partner smoking at 4 years**				0.562
No	771 (65.0)	82 (6.9)	334 (28.1)	
Yes	1,099 (65.0)	134 (7.9)	461 (27.2)	
**1993 Cohort**				
**Familiar income (tertile)**				< 0.0001
1st	978 (62.7)	67 (4.3)	514 (33.0)	
2nd	727 (66.4)	49 (4.5)	319 (29.1)	
3rd	813 (74.9)	49 (4.5)	224 (20.6)	
**Maternal schooling (years)**				< 0.0001
0 to 4	576 (57.1)	52 (5.1)	381 (37.8)	
5 to 8	1,185 (66.4)	86 (4.8)	513 (28.8)	
≥ 9	802 (79.2)	31 (3.1)	179 (17.7)	
**Partner smoking in pregnancy**				< 0.0001
No	1,318 (77.9)	100 (5.9)	274 (16.2)	
Yes	1,396 (58.0)	226 (9.4)	785 (32.6)	
