# Supplementary material for: Maternal smoking during pregnancy and offspring body composition in adulthood: Results from two birth cohort studies
Source: BMJ Open. 2019 Jun 12;9(6):e023852. doi: 10.1136/bmjopen-2018-023852 (PMC6575638; doi:10.1136/bmjopen-2018-023852)
Supplement: Author's manuscript [file bmjopen-2018-023852.draft_revisions.pdf]

BMJ Open

BMJ Open is committed to open peer review. As part of this commitment we make the peer review history of every article we publish publicly available.

When an article is published we post the peer reviewers' comments and the authors' responses online. We also post the versions of the paper that were used during peer review. These are the versions that the peer review comments apply to.

The versions of the paper that follow are the versions that were submitted during the peer review process. They are not the versions of record or the final published versions. They should not be cited or distributed as the published version of this manuscript.

BMJ Open is an open access journal and the full, final, typeset and author-corrected version of record of the manuscript is available on our site with no access controls, subscription charges or pay-per-view fees (http://bmjopen.bmj.com).

If you have any questions on BMJ Open's open peer review process please email info.bmjopen@bmj.com

BMJ Open

Maternal smoking during pregnancy and offspring body composition in adulthood: Results from two birth cohort studies

Journal:	*BMJ Open*
Manuscript ID	bmjopen-2018-023852
Article Type:	Research
Date Submitted by the Author:	28-Apr-2018
Complete List of Authors:	Magalhães, Elma Izze; Universidade Federal de Pelotas, Programa de Pós-Graduação em Epidemiologia<br>Lima, Natália; Universidade Federal de Pelotas, Programa de Pós-Graduação em Epidemiologia<br>Menezes, Ana Maria ; Universidade Federal de Pelotas, Programa de Pós-Graduação em Epidemiologia<br>Goncalves, Helen; Universidade Federal de Pelotas, Programa de Pós-Graduação em Epidemiologia<br>Wehrmeister, Fernando; Universidade Federal de Pelotas, Programa de Pós-Graduação em Epidemiologia<br>Assuncao, M.C; Universidade Federal de Pelotas, Programa de Pós-Graduação em Epidemiologia<br>Horta, Bernardo; Federal University of Pelotas, Postgraduate Program in Epidemiology
Keywords:	Maternal smoking, Pregnancy, Body Composition, Cohort study

SCHOLARONE™
Manuscripts

For peer review only

Maternal smoking during pregnancy and offspring body composition in adulthood: Results from two birth cohort studies

Elma Izze da Silva Magalhães[1] — elmaizzenutri@gmail.com

Natália Peixoto Lima[1] — natyplima@hotmail.com

Ana Maria Baptista Menezes[1] — anamene.epi@gmail.com

Helen Gonçalves[1] — hdgs.epi@gmail.com

Fernando C. Wehrmeister[1] — fcwehrmeister@gmail.com.br

Maria Cecília Formoso Assunção[1] — cecilia.epi@gmail.com

Bernardo Lessa Horta[1] — blhorta@gmail.com

[1]Postgraduate Programme in Epidemiology, Federal University of Pelotas, Pelotas, Rio Grande do Sul, Brazil.

Correspondence to:

Elma Izze da Silva Magalhães. Postgraduate Programme in Epidemiology, Rua Marechal Deodoro, 1160, 3º piso, Centro, Pelotas, RS, Brazil. Cep: 96020-220 - Caixa Postal 464. Telephone number: +55 53 98159-5411. E-mail address: elma_izze@hotmail.com.

Word count: 2,667

For peer review only - http://bmjopen.bmj.com/site/about/guidelines.xhtml

ABSTRACT

Objective: To evaluate the association of maternal smoking during pregnancy with offspring body composition in adulthood and explore the causality of this association.

Design: Birth cohort.

Setting: Population-based study in Pelotas, Brazil.

Participants: All newborn infants in the city's hospitals were enrolled in 1982 and 1993. At 30 and 22 years, the 1982 and 1993 cohorts, respectively, followed the subjects and 7,222 subjects were evaluated.

Primary outcome measures: Body mass index, fat mass index, android to gynoid fat ratio, waist circumference, waist to height ratio, lean mass index and height.

Results: Prevalence of maternal smoking during pregnancy was 35.1% and 32.6%, in 1982 and 1993 cohorts, respectively. Offspring of smoking mothers showed higher mean body mass index (β: 0.84; 95%CI: 0.55 to 1.12), fat mass index (β: 0.44; 95%CI: 0.23 to 0.64), android to gynoid fat ratio (β: 0.016; 95%CI: 0.010 to 0.023), waist circumference (β: 1.74; 95%CI: 1.15 to 2.33), waist to height ratio (β: 0.013; 95%CI: 0.010 to 0.017) and lean mass index (β: 0.33; 95%CI: 0.24 to 0.42), whereas height was lower (β: -0.95; -1.26 to -0.65). Weight gain in the first two years captured most of the association of maternal smoking with body mass index (96.2%), waist circumference (86.1%) and fat mass index (71.7%).

Conclusions: Maternal smoking in pregnancy was associated with offspring body composition measures in adulthood.

Keywords: Maternal smoking; Pregnancy; Body Composition; Cohort study.

For peer review only

ARTICLE SUMMARY

Strengths and limitations of this study

- High follow-up rate, and the lack of differences in the follow-up rate according to maternal smoking during pregnancy, suggesting that selection bias is unlikely.
- We increased causal inference by comparing the estimates of maternal and paternal smoking.
- Body composition was assessed using dual X-ray absorptiometry and air-displacement pletysmography.
- We evaluated smoking in pregnancy using a questionnaire and the information was not validated with biochemical markers.
- Information on partner smoking was obtained at different times in the two cohorts.

For peer review only
For peer review only - http://bmjopen.bmj.com/site/about/guidelines.xhtml

INTRODUCTION

Maternal smoking during pregnancy has clear short-term consequences, increasing the risk of low birthweight and intrauterine growth retardation.[1] Moreover, maternal smoking is also associated with increased infant mortality rate, and cessation of smoking during pregnancy reduces the risk of infant death.[2] However, evidence on the long-term consequences of maternal smoking are not clear-cut. Meta-analysis by Brion et al.[3] related that blood pressure was slightly higher among offspring of smoking mothers. It has also been reported a higher risk of diabetes.[4] On the other hand, Horta et al.[5] observed that the association between maternal smoking in the pregnancy and metabolic cardiovascular risk factors in early adulthood was mediated by offspring lifestyle in adulthood.

With respect to body composition, it has been suggested that maternal smoking in pregnancy is positively associated with adiposity and risk of overweight/obesity in the offspring.[6, 7] On the other hand, because socioeconomic status is negatively associated with maternal smoking[8] and obesity,[9] the association of maternal smoking with offspring obesity could be due to residual confounding.[10] Indeed, some studies have reported that after controlling for socioeconomic and environmental variables, the magnitude of the association decreased.[11, 12]

Because socioeconomic status is usually assessed using few variables, the full dimension of socioeconomic status may not have been captured. And, residual confounding may be observed even among those studies that adjusted the estimates to socioeconomic and demographic variables. Comparison of the magnitude of the associations with maternal and paternal smoking is a strategy that can be used to increase causal inference. Similar associations for paternal and maternal smoking would suggest that confounding by socioeconomic, environmental or familiar variables is an explanation for the observed

For peer review only

For peer review only - http://bmjopen.bmj.com/site/about/guidelines.xhtml

associations. On the other hand, if the association is stronger for maternal variables, it is unlikely that the observed association is due to residual confounding.[13]

This study was aimed at assessing the association of maternal smoking during pregnancy with offspring body composition in adulthood and explore the causality of this association.

METHODS

Study Design and Population

This study is based on data from two birth cohorts carried out in Pelotas, a southern Brazilian city. In 1982 and 1993, in the perinatal study, all maternity hospitals in the city were visited daily, and the births identified. Those liveborns whose families lived in the urban area of the city were examined and their mothers interviewed soon after delivery. These subjects have been followed-up for several times through the life cycle, and further details on the study methodology have been published elsewhere.[14, 15]

Exposure Variables

In both cohorts, information on maternal smoking during pregnancy, duration and numbers of cigarettes smoked per day were obtained from the mothers in the perinatal study. Those mothers who reported any tobacco use during pregnancy were considered as smokers, and they were asked whether they had smoked during the whole pregnancy and how many cigarettes on average they had smoked per day. In addition, we also collected information on partner smoking. In the 1993 cohort, this information was gathered in the perinatal study, whereas in the 1982 cohort, information on partner smoking was provided by the mother in the follow-up visit at 4 years of age.

For peer review only - http://bmjopen.bmj.com/site/about/guidelines.xhtml

Outcome Variables

From June 2012 to February 2013 and October 2015 to July 2016, the participants of the 1982 and 1993 cohort, respectively, were invited to visit the research clinic to be interviewed, examined and donate a blood sample. Weight was measured using the Bod Pod scale and height with a portable stadiometer (accuracy of 0.1 cm). Body mass index (BMI) was estimated by dividing weight (in kg) by square height (in meters).

Waist circumference (in cm) was measured with the subject standing, with the arms hanging freely and next to the body, using a non-elastic measuring tape in the horizontal plane around the narrowest part of the waist. In obese subjects, the measure was taken in the horizontal plane at the point between the last rib and the iliac crest. Waist to height ratio was estimated by dividing waist circumference by height (both in cm).

Fat-free and fat mass (total and in the android and gynoid regions) were assessed using dual-energy x-ray absorptiometry (DXA). Lean and fat mass indexes were estimated by dividing fat-free and fat mass (in kg) by square height in meters. Android to gynoid fat ratio was estimated by dividing percentage fat in region android by percentage fat in gynoid region. Pregnant women or those in the postpartum period (three months) were excluded from this assessment.

Confounders

The following variables were considered as possible confounders: sex, age at assessment of the outcomes, family income at birth (total income earned by the family members in the month before the interview), maternal skin colour (self-reported), maternal schooling (years of schooling successfully completed), maternal age at childbirth, parity (number of previous deliveries), pre-gestational nutritional status (height was measured by the

For peer review only

research team and information on prepregnancy maternal weight was obtained from the antenatal care records or by maternal recall).

Mediators

Birthweight and weight gain from birth to two years, were considered as possible mediators. Birthweight (in grams) was assessed soon after delivery, by the hospital staff, using paediatric scales that were calibrated weekly by the research team. Weight gain from birth to two years of age was obtained by the difference in z-score between the child's weight at 2 years of age and the birthweight. The weight of the child at two years of age was measured only in the follow-up of the 1982 cohort. Thus, this analysis was only performed only for the 1982 cohort.

Statistical Analysis

Stata, version 14.0 (StataCorp, College Station, TX) was used in the analysis. Data from the two cohorts were analysed together, because the magnitude of the association of maternal smoking with offspring body composition was not modified by cohort (data not shown). Means were compared using Student t-test and analysis of variance (ANOVA). Multiple linear regression was used to obtain estimates that were adjusted for confounding variables. G-computation (bootstrap replications: 10,000) was used to estimate direct and indirect effects of maternal smoking in pregnancy on offspring body composition in adulthood. In the analysis for birthweight, gestational age was considered as post-confounder, while for weight gain from birth to two years, we considered family income in childhood as a post-confounder. Sex, age at body composition assessment, family income at birth, parity,

For peer review only - http://bmjopen.bmj.com/site/about/guidelines.xhtml

pre-gestational BMI, skin colour, maternal schooling, and height were included as base-confounders.

Patient and Public Involvement

The study was approved by the Ethical Review Board of the Faculty of Medicine of the Federal University of Pelotas, and was performed in accordance with the ethical standards established in the 1964 Declaration of Helsinki and its later amendments. Written informed consent was obtained from participating subjects.

RESULTS

From June 2012 and February 2013, 3,701 participants of the 1982 cohort were interviewed, which added to the 325 deaths identified among the cohort participants, represented a follow-up rate of 68.1%. In 2015-16, 3,810 participants of the 1993 cohort were examined, which represented a follow-up rate of 76.3%, after taking into consideration the 193 deaths identified among the cohort members.[14, 15] Information on maternal smoking and at least one of the outcomes were available for 3,626 participants from 1982 cohort and 3,596 from 1993.

Table 1 shows the characteristics of the subjects included in the analyses. The proportion of preterm births was 5.6% and 8.0% in 1982 and 1993, respectively. The prevalence of overweight/obesity was 43.2% at 22 years (1993 cohort) and 57.6% at 30 years (1982 cohort). Prevalence maternal smoking during pregnancy was 35.0% and 32.4% in 1982 and 1993, respectively. Moreover, 32.4% of the partners smoked during pregnancy in the 1993 cohort and 58.8% of the partners were smoker at the follow-up visit at 4 years of age.

In the crude analyses (Table 2), maternal smoking in the pregnancy was associated with higher mean BMI, fat mass index, android to gynoid fat ratio, waist circumference, waist

For peer review only - http://bmjopen.bmj.com/site/about/guidelines.xhtml

to height ratio, as well as, lean mass index. On the other hand, offspring of mothers who smoked during pregnancy had lower height. With respect to duration of maternal smoking during pregnancy, body mass, fat mass index, android to gynoid fat ratio, waist circumference, waist to height ratio and lean mass index were slightly higher among the offspring of mothers who smoked only in part of pregnancy in relation to those whose mother smoked in the whole pregnancy. But the lower means were observed among those subjects whose mothers did not smoke in the pregnancy. On the other hand, height was inversely associated with the duration of maternal smoking during pregnancy.

After controlling for confounding variables, offspring of smoking mothers showed higher body mass index, fat mass index, android to gynoid fat ratio, waist circumference, waist to height ratio and lean mass index, whereas height was lower. Concerning, the duration and intensity of maternal smoking during pregnancy, we only observed a dose response association for height, whereas for the remaining body composition outcomes, the regression coefficients tend to slightly higher among those subjects whose mother stopped smoking in the pregnancy or smoked from 1 to 14 cigarettes/day. (Table 3).

Table 4 shows that maternal smoking was associated with most of the body composition outcomes, even after controlling for partner smoking. Moreover, in both cohorts the magnitude of the associations were stronger for maternal than partner smoking. Because information on partner smoking during pregnancy was not available for the 1982 cohort, we used the information on partner smoking collected in the follow-up visit at 4 years of age.

Concerning the mediation analysis, birthweight captured a small portion of the associations, whereas weight gain in the first two years captured most of the association of maternal smoking with body mass index, fat mass index and waist circumference (Table 5).

For peer review only

For peer review only - http://bmjopen.bmj.com/site/about/guidelines.xhtml

DISCUSSION

Our findings suggest that exposure to maternal tobacco smoking in utero increases adiposity in early adulthood. These associations were observed even after controlling for several confounding variables. Furthermore, we observed that the magnitude of the association was higher for maternal than paternal smoking and even after controlling for paternal smoking, maternal smoking during pregnancy was associated with body composition in adulthood. Suggesting, therefore, that residual confounding is an unlikely explanation for the association between maternal smoking and offspring body composition.

The results of our study corroborate the findings from previous studies that have observed a positive association between maternal smoking during pregnancy and offspring adiposity.[11, 12, 16] In relation to central adiposity, it has also been reported higher means waist circumference[16] and android to gynoid fat ratio.[17] Such measures are indicators of fat accumulation in the abdominal region and are more related to cardiovascular risk.[18]

Concerning the mechanisms underlying the association between maternal smoking during pregnancy and offspring adiposity, it has been suggested that this association is due to the nicotine, present in tobacco. In both humans and animals, nicotine when crossing the placenta acts centrally and peripherally as a suppressant of appetite and body weight, resulting in hyperphagia and weight gain when the offspring is no longer exposed to nicotine in postnatal period.[19, 20] In addition, exposure to nicotine in gestation may result in increase in body adiposity through alterations in endocrine control of body weight homeostasis.[21] In animals, prenatal exposure to nicotine decreased responsiveness to adrenergic stimuli and promoted rapid weight gain.[22] Similarly, prenatal exposure to nicotine in humans may decrease responsiveness to adrenergic stimuli by epinephrine and norepinephrine, which modulate the mobilization of lipids from adipose tissue.[23]

For peer review only - http://bmjopen.bmj.com/site/about/guidelines.xhtml

It has been reported that low birthweight is associated with increased risk of obesity later in life,[24, 25] but in the present study we did not find any evidence that birthweight mediated the observed associations. On the other hand, weight gain in early childhood captured an important proportion of the association of maternal smoking in pregnancy with BMI, fat mass index and waist circumference. Maternal smoking tends to decrease foetal growth, which would be compensated by rapid postnatal weight gain.[26] Because offspring of smoking mothers were shorter, it seems that weight gain was higher than linear growth. Indeed, Adair et al.[27] reported that faster relative weight gain in the first two years was associated with a higher risk of overweight/obesity in adulthood.

In our study, we observed that lean mass was higher among offspring of mothers who smoked in pregnancy. Leary et al.[11] also observed that lean mass was higher among subjects whose mother smoked in the pregnancy. These authors suggested that this association with lean mass would be simply due to the association with fat mass. Indeed, subjects with higher total body mass would have higher fat and lean mass. Concerning the association with height, a negative association has already been reported by previous studies.[12, 28]

Our study has many strengths, the data was collected prospectively and information on maternal smoking during pregnancy was collected soon after delivery, with a short recall time. Previous studies on this subject are mainly retrospective, or cross-sectional,[28, 29] and data on maternal smoking was gathered with a long recall time, which can lead to misclassification. Secondly, the high follow-up rates, even after 30 years and the lack of differences in the follow-up rate according to maternal smoking during pregnancy suggests that selection bias is unlikely.[14] Finally, most of the previous studies have evaluated only BMI as a measure of adiposity. We used several anthropometric and body composition measurements, including fat and lean mass measured using DXA. By comparing the estimates

For peer review only - http://bmjopen.bmj.com/site/about/guidelines.xhtml

of maternal smoking to partner smoking, we increased causal inference and the analysis suggested that the observed associations were unlikely to be due to residual confounding.

With respect to the limitations, information on parental smoking was gathered using a questionnaire and the information was not validated with biochemical markers. Because smoking in pregnancy is a socially disapproved act, some mothers may have occulted the information on prenatal smoking,[30] underestimating the prevalence of smoking during pregnancy. Because this classification error was independent of body composition, which was measured many years later, the misclassification would be non-differential. Because non-differential misclassification tends to underestimate the magnitude of the associations, the observed associations are not due to such error. Another limitation is the fact that information on partner smoking was obtained at different times in the two cohorts. However, it is likely that parents who smoked during pregnancy remained smokers after birth and the first years of the offspring's life. In addition, no discrepant results were observed regarding the effect of paternal smoking among the two cohorts.

In conclusion, we observed an association between maternal smoking in pregnancy and body composition measures in adulthood. The specificity of the association for maternal, in relation to paternal smoking, suggested that this association is not due to residual confounding. Analysis of mediation suggest the weight gain from birth to two years is an important mediator of the association between maternal smoking during pregnancy and adiposity in adulthood.

CONTRIBUTORSHIP STATEMENT

EISM designed the study, performed the statistical analysis, interpretation of the results and drafted the manuscript. BLH and NPL designed the study, helped the data

For peer review only - http://bmjopen.bmj.com/site/about/guidelines.xhtml

analysis, and participated in the preparation of the manuscript. BLH coordinated the follow-up of the 1982 cohort and AMBM coordinated the follow-up of the 1993 cohort. AMBM, HDG, FCW, and MCA helped in the data acquisition and interpretation of the data. All authors revised and approved the final version of the manuscript. Each author contributed important intellectual content during manuscript drafting or revision and accepts accountability for the overall work by ensuring that questions pertaining to the accuracy or integrity of any portion of the work are appropriately investigated and resolved.

COMPETING INTERESTS

None declared.

FUNDING

This article is based on data from the study "Pelotas Birth Cohort, 1982 and 1993" conducted by the Postgraduate Program in Epidemiology at Federal University of Pelotas with the collaboration of the Brazilian Public Health Association (ABRASCO). From 2004 to 2016, the Wellcome Trust (086974/Z/08/Z) supported the Pelotas birth cohort study. The International Development Research Center, World Health Organization, Overseas Development Administration, European Union, National Support Program for Centers of Excellence (PRONEX), the Brazilian National Research Council (CNPq), and the Brazilian Ministry of Health supported previous phases of the study.

DATA SHARING STATEMENT

Data are available through the Centre of Epidemiological Research and the Postgraduate Program in Epidemiology.

For peer review only

For peer review only - http://bmjopen.bmj.com/site/about/guidelines.xhtml

ACKNOWLEDGEMENTS

We acknowledge the contributions of 1982 and 1993 Pelotas Cohorts participants, others researchers and staff.

For peer review only - http://bmjopen.bmj.com/site/about/guidelines.xhtml

REFERENCES

1. Kramer MS. Determinants of low birthweight: methodological assessment and meta-analysis. *Bull World Health Organ* 1987;65:663–737.

2. Johansson ALV, Dickman PW, Kramer MS, et al. Maternal smoking and infant mortality: does quitting smoking reduce the risk of infant death?. *Epidemiology* 2009;20:590–597.

3. Brion MJA, Leary SD, Lawlor DA, et al AR. Modifiable maternal exposures and offspring blood pressure: a review of epidemiological studies of maternal age, diet, and smoking. *Pediatr Res* 2008; 63:593–598.

4. Montgomery SM, Ekbom A. Smoking during pregnancy and diabetes mellitus in a British longitudinal birth cohort. *BMJ* 2002;324:26–27.

5. Horta BL, Gigante DP, Nazmi A, et al. Maternal smoking during pregnancy and risk factors for cardiovascular disease in adulthood. *Atherosclerosis* 2011;219:815–820.

6. Oken E, Levitan EB, Gillman MW. Maternal smoking during pregnancy and child overweight: systematic review and meta-analysis. *Int J Obes* 2008; 32:201–210.

7. Ino T. Maternal smoking during pregnancy and offspring obesity: Meta-analysis. *Pediatr Int* 2010; 52:94–99.

For peer review only

8. Iliadou AN, Koupil I, Villamor E, Altman D, Hultman C, Langström N, et al. Familial factors confound the association between maternal smoking during pregnancy and young adult offspring overweight. *Int J Epidemiol* 2010;39:1193–1202.

9. Monteiro CA, Moura EC, Conde WL, et al. Socioeconomic status and obesity in adult populations of developing countries: a review. *Bull World Health Organ* 2004;82:940–946.

10. Riedel C, Schönberger K, Yang S, et al. Parental smoking and childhood obesity: higher effect estimates for maternal smoking in pregnancy compared with paternal smoking—a meta-analysis. *Int J Epidemiol* 2014;43:1593–1606.

11. Leary SD, Smith GD, Rogers IS, et al. Smoking during pregnancy and offspring fat and lean mass in childhood. *Obesity* 2006;14:2284–2293.

12. Chen A, Pennell ML, Klebanoff MA, et al. Maternal smoking during pregnancy in relation to children overweight: Follow-up to age 8 years. *Int J Epidemiol* 2006;35:121–130.

13. Howe LD, Matijasevich A, Tilling K, et al. Maternal smoking during pregnancy and offspring trajectories of height and adiposity: comparing maternal and paternal associations. *Int J Epidemiol* 2012;41:722–732.

14. Horta BL, Gigante DP, Gonçalves H, et al. Cohort profile update: the 1982 Pelotas (Brazil) birth cohort study. *Int J Epidemiol* 2015;44:441–441e.

For peer review only - http://bmjopen.bmj.com/site/about/guidelines.xhtml

15. Gonçalves H, Wehrmeister FC, Assunção MC, et al. Cohort Profile Update: The 1993 Pelotas (Brazil) Birth Cohort follow-up at 22 years. *Int J Epidemiol* 2017;1–7.

16. Mamun AA, O'Callaghan MJ, Williams GM, et al. Maternal smoking during pregnancy predicts adult offspring cardiovascular risk factors-evidence from a community-based large birth cohort study. *PloS One* 2012;7:e41106.

17. Durmuş B, Heppe DHM, Taal HR, et al. Parental smoking during pregnancy and total and abdominal fat distribution in school-age children: the Generation R Study. *Int J Ob* 2014;38:966–972.

18. Lee CMY, Huxley RR, Wildman RP, et al. Indices of abdominal obesity are better discriminators of cardiovascular risk factors than BMI: a meta-analysis. *J Clin Epidemiol* 2008;61:646–653.

19. Li MD, Parker SL, Kane JK. Regulation of feeding-associated peptides and receptors by nicotine. *Mol Neurobiol* 2000;22:143–165.

20. Jo YH, Talmage DA, Role LW. Nicotinic receptor□mediated effects on appetite and food intake. *J Neurobiol* 2002;53:618–632.

21. Bruin JE, Gerstein HC, Holloway AC. Long-term consequences of fetal and neonatal nicotine exposure: a critical review. *Toxicol Sci* 2010;116:364–374.

For peer review only - http://bmjopen.bmj.com/site/about/guidelines.xhtml

22. Levin ED. Fetal nicotinic overload, blunted sympathetic responsivity, and obesity. *Birth Defects Res A Clin Mol Teratol* 2005;73:481–484.

23. Schiffelers SLH, Saris WHM, Boomsma F, et al. β1-and β 2-Adrenoceptor-Mediated Thermogenesis and Lipid Utilization in Obese and Lean Men. *J Clin Endocrinol Metab* 2001;86:2191–2199.

24. Malina, RM, Katzmarzyk PT, Beunen G. Birthweight and its relationship to size attained and relative fat distribution at 7 to 12 years of age. *Obesity* 1996; 4:385–390.

25. Singhal A, Wells J, Cole TJ, et al. Programming of lean body mass: a link between birthweight, obesity, and cardiovascular disease?. *Am J Clin Nutr* 2003;77:726–730.

26. Oken E, Gillman MW. Fetal origins of obesity. *Obesity* 2003;11:496–506.

27. Adair LS, Fall CH, Osmond C, et al. Associations of linear growth and relative weight gain during early life with adult health and human capital in countries of low and middle income: findings from five birth cohort studies. *Lancet* 2013;382:525–534.

28. Koshy G, Delpisheh A, Brabin BJ. Dose response association of pregnancy cigarette smoke exposure, childhood stature, overweight and obesity. *Eur J Public Health* 2011; 21:286–291.

For peer review only - http://bmjopen.bmj.com/site/about/guidelines.xhtml

29. Adams AK, Harvey HE, Prince R.J. Association of maternal smoking with overweight at age 3 y in American Indian children. *Am J Clin Nutr* 2005; 82:393–398.

30. Dietz PM, Homa D, England LJ, et al. Estimates of nondisclosure of cigarette smoking among pregnant and nonpregnant women of reproductive age in the United States. *Am J Epidemiol* 2011; 173:355–359.

For peer review only

Variable	1982 Cohort (N= 3,626)		1993 Cohort (N= 3,596)	
	N [a] (%)	Mean (SD)	N [a] (%)	Mean (SD)
**Sex**				
Male	1,766 (48.7)		1,706 (47.4)	
Female	1,860 (51.3)		1,890 (52.6)	

For peer review only

Table 1. Characteristics of population included in the present study, Pelotas, Brazil.

For peer review only - http://bmjopen.bmj.com/site/about/guidelines.xhtml

**Gestational age (weeks)**				
< 37	164 (5.6)		283 (8.0)	
37 to 39	1,336 (45.8)		2,741 (77.2)	
≥ 40	1,420 (48.6)		527 (14.8)	
**Birth weight (g)**				
< 2,500	280 (7.7)		338 (9.4)	
2,500 a 2,999	956 (26.4)		942 (26.2)	
≥ 3,000	2,389 (65.9)		2,314 (64.4)	
**Skin colour**				
White	2,750 (75.8)		2,163 (63.5)	
Non-white	876 (24.2)		1,245 (36.5)	
**Maternal smoking during pregnancy**				
Non smokers	2,353 (65.0)		2,430 (67.6)	
Smoked in part of pregnancy	276 (7.6)		160 (4.4)	
Smoked in whole pregnancy	990 (27.4)		1,006 (28.0)	
**Partner smoking**				
No	1,166 (41.2)		2,433 (67.6)	
Yes	1,665 (58.8)		1,163 (32.4)	
**Body mass index in adulthood (kg/m^2)**		26.8 (5.5)		25.2 (5.3)
< 18.5	71 (2.0)		168 (4.7)	
18.5 to 24.9	1,426 (40.4)		1,855 (52.1)	
25.0 to 29.9	1,224 (34.7)		959 (27.0)	
≥ 30.0	810 (22.9)		577 (16.2)	
**Fat mass index (kg/m^2)**		8.7 (4.3)		7.9 (4.5)
**Android to gynoid fat ratio**		0.50 (0.14)		0.42 (0.12)
**Waist circumference (cm)**		84.8 (12.6)		80.0 (11.6)
**Waist to height ratio**		0.51 (0.07)		0.48 (0.07)
**Lean mass index (kg/m^2)**		16.7 (2.7)		16.1 (2.6)
**Height (cm)**		167.7 (9.2)		167.4 (9.5)

Abbreviation: SD, standard deviation; IQI, inter quartiles interval.
[a] The total number of subjects per variable may not add up to 3,626 (1982 cohort) or 3,596 (1993 cohort) due to missing data.
[b] At 4 years for the 1982 cohort and in pregnancy for the 1993 cohort.

For peer review only

For peer review only - http://bmjopen.bmj.com/site/about/guidelines.xhtml

Table 2. Mean for anthropometric and body composition measures in adulthood and according to maternal smoking during pregnancy.

	Mean (Standard deviation)						
	**Body mass index (kg/m^2)**	**Fat mass index (kg/m^2)**	**Android to gynoid fat ratio**	**Waist circumference (cm)**	**Waist to height ratio**	**Lean mass index (kg/m^2)**	**Height (cm)**
**Total**	26.0 (5.5)	8.3 (4.4)	0.46 (0.14)	82.4 (12.4)	0.51 (0.07)	16.7 (2.7)	167.7 (9.2)
**Maternal smoking in pregnancy [a]**	*P=<0.0001* ****	*P=0.0090* **	*P=<0.0001* ****	*P=0.0001* ****	*P=0.0001* ****	*P=0.0001* ****	*P=0.0001* ****

[a] Student t-test
[b] Analysis of variance (ANOVA)
*P<0.05; **P<0.01; ****P<0.0001.

For peer review only - http://bmjopen.bmj.com/site/about/guidelines.xhtml

No	25.8 (5.4)	8.2 (4.3)	0.46 (0.13)	82.0 (12.3)	0.49 (0.07)	16.3 (2.6)	168.1 (9.4)
Yes	26.4 (5.6)	8.5 (4.6)	0.47 (0.14)	83.2 (12.5)	0.50 (0.07)	16.6 (2.6)	166.6 (9.3)
**Duration of maternal smoking** [b]	*P=<0.0001*[****]	*P=0.0036*[**]	*P=<0.0001*[****]	*P=<0.0001*[****]	*P=<0.0001*[****]	*P=<0.0001*[****]	*P=<0.0001*[****]
Non smokers	25.8 (5.4)	8.2 (4.3)	0.46 (0.13)	82.0 (12.3)	0.49 (0.07)	16.3 (2.6)	168.1 (9.4)
Smoked in part of pregnancy	27.0 (5.6)	9.0 (4.6)	0.48 (0.13)	84.7 (12.5)	0.50 (0.07)	16.7 (2.6)	167.8 (9.6)
Smoked in whole pregnancy	26.3 (5.6)	8.4 (4.6)	0.47 (0.14)	82.9 (12.4)	0.50 (0.07)	16.6 (2.6)	166.3 (9.2)
**Cigarettes smoked per day** [b]	*P=0.0001*[****]	*P=0.0165*[*]	*P=<0.0001*[****]	*P=0.0007* [b]	*P=<0.0001*[****]	*P=<0.0001*[****]	*P=<0.0001*[****]
None	25.8 (5.4)	8.2 (4.3)	0.46 (0.13)	82.0 (12.3)	0.49 (0.07)	16.3 (2.6)	168.1 (9.4)
1 to 14 cigarettes/day	26.5 (5.6)	8.6 (4.6)	0.47 (0.14)	83.2 (12.5)	0.50 (0.07)	16.6 (2.6)	166.7 9.3)
≥ 15 cigarettes/day	26.3 (5.4)	8.3 (4.5)	0.48 (0.14)	83.2 (12.5)	0.50 (0.07)	16.7 (2.5)	165.9 (9.4)

Table 3. Adjusted regression coefficients for anthropometric and body composition measures in adulthood according to maternal smoking during pregnancy.

	**Adjusted regression coefficient (95% confidence interval)** [a]						
	**Body mass index (kg/m^2)**	**Fat mass index (kg/m^2)**	**Android to gynoid fat ratio**	**Waist circumference (cm)**	**Waist to height ratio**	**Lean mass index (kg/m^2)**	**Height (cm)**
**Maternal smoking in pregnancy**							
No	Reference	Reference	Reference	Reference	Reference	Reference	Reference
Yes	0.84	0.44	0.016	1.74	0.013	0.33	-0.95
	(0.55 ; 1.12)	(0.23 ; 0.64)	(0.010 ; 0.023)	(1.15 ; 2.33)	(0.010 ; 0.017)	(0.24 ; 0.42)	(-1.26 ; -0.65)
**Duration of maternal smoking**							
Non-smokers	Reference	Reference	Reference	Reference	Reference	Reference	Reference
Smoked in part of pregnancy	1.17	0.70	0.013	2.49	0.016	0.34	-0.43
	(0.62 ; 1.71)	(0.30 ; 1.11)	(0.001 ; 0.026)	(1.32 ; 3.65)	(0.009 ; 0.023)	(0.16 ; 0.53)	(-1.03 ; 0.18)
Smoked in whole Pregnancy	0.76	0.37	0.017	1.56	0.013	0.32	-1.07

For peer review only - http://bmjopen.bmj.com/site/about/guidelines.xhtml

	(0.46 ; 1.06)	(0.15 ; 0.59)	(0.010 ; 0.024)	(0.93 ; 2.19)	(0.009 ; 0.016)	(0.22 ; 0.42)	(-1.40 ; -0.74)
**Cigarettes smoked per day**							
None	Reference	Reference	Reference	Reference	Reference	Reference	Reference
1 to 14 cigarettes/day	0.89<br>(0.60 ; 1.19)	0.46<br>(0.24 ; 0.69)	0.018<br>(0.011 ; 0.025)	1.86<br>(1.23 ; 2.50)	0.014<br>(0.010 ; 0.017)	0.34<br>(0.24 ; 0.44)	-0.81<br>(-1.15 ; -0.49)
≥ 15 cigarettes/day	0.62<br>(0.09 ; 1.15)	0.32<br>(-0.08 ; 0.71)	0.010<br>(-0.003 ;0.022)	1.24<br>(0.12 ; 2.36)	0.012<br>(0.005 ; 0.019)	0.30<br>(0.12 ; 0.47)	-1.52<br>(-2.11 ; -0.94)

[a] Adjusted for: Sex, age (Cohort), family income at birth, maternal skin colour, maternal schooling, maternal age at childbirth, parity, pre-gestational nutritional status and maternal height.

Table 4. Adjusted regression coefficients for anthropometric and body composition measures in adulthood according to maternal smoking during pregnancy and partner smoking.

	Adjusted regression coefficient (95% confidence interval)						
	**Body mass index (kg/m^2)**	**Fat mass index (kg/m^2)**	**Android to gynoid fat ratio**	**Waist circumference (cm)**	**Waist to height ratio**	**Lean mass index (kg/m^2)**	**Height (cm)**
**1982 cohort**							
**Maternal smoking in pregnancy** [a]							
No	Reference	Reference	Reference	Reference	Reference	Reference	Reference
Yes	0.65<br>(0.24 ; 1.06)	0.30<br>(0.01 ; 0.59)	0.020<br>(0.011 ; 0.030)	1.54<br>(0.67 ; 2.41)	0.012<br>(0.006 ; 0.017)	0.27<br>(0.13 ; 0.41)	-0.75<br>(-1.17 ; -0.33)
**Partner smoking at 4 years** [a]							
No	Reference	Reference	Reference	Reference	Reference	Reference	Reference
Yes	-0.04<br>(-0.47 ; 0.39)	0.01<br>(-0.29 ; 0.33)	0.001<br>(-0.010 ; 0.011)	-0.05<br>(-0.97 ; 0.88)	0.001<br>(-0.005 ; 0.006)	0.02<br>(-0.12 ; 0.17)	-0.11<br>(-0.56 ; 0.34)

For peer review only

**Maternal smoking in pregnancy [b]**							
No	Reference	Reference	Reference	Reference	Reference	Reference	Reference
Yes	0.64	0.24	0.016	1.60	0.012	0.24	-0.73
	(0.19 ; 1.10)	(-0.09 ; 0.56)	(0.005 ;0.027)	(0.62 ; 2.57)	(0.006 ; 0.018)	(0.09 ; 0.39)	(-1.21 ; -0.26)
**Partner smoking at 4 years [c]**							
No	Reference	Reference	Reference	Reference	Reference	Reference	Reference
Yes	-0.04	0.02	0.001	-0.05	0.001	0.02	-0.11
	(-0.47 ; 0.39)	(-0.29 ; 0.33)	(-0.010 ; 0.011)	(-0.97 ; 0.87)	(-0.005 ; 0.006)	(-0.12 ; 0.17)	(-0.55 ; 0.35)
**1993 cohort**							
**Maternal smoking in pregnancy [a]**							
No	Reference	Reference	Reference	Reference	Reference	Reference	Reference
Yes	1.04	0.38	0.014	1.99	0.016	0.38	-1.21
	(0.66 ; 1.42)	(0.25 ; 0.51)	(0.006 ; 0.023)	(1.18 ; 2.80)	(0.011 ; 0.021)	(0.25 ; 0.51)	(-1.66 ; -0.76)
**Partner smoking in pregnancy [a]**							
No	Reference	Reference	Reference	Reference	Reference	Reference	Reference
Yes	0.48	0.20	0.006	1.23	0.009	0.21	-0.28
	(0.11 ; 0.85)	(-0.09 ; 0.48)	(-0.002 ; 0.014)	(0.44 ; 2.01)	(0.004 ; 0.013)	(0.09 ; 0.34)	(-0.71 ; 0.15)
**Maternal smoking in pregnancy [d]**							
No	Reference	Reference	Reference	Reference	Reference	Reference	Reference
Yes	1.01	0.36	0.015	1.92	0.015	0.36	-1.13
	(0.61 ; 1.42)	(0.23 ; 0.50)	(0.006 ;0.024)	(1.05 ; 2.78)	(0.009 ; 0.020)	(0.23 ; 0.50)	(-1.61 ; -0.65)
**Partner smoking in pregnancy [c]**							
No	Reference	Reference	Reference	Reference	Reference	Reference	Reference
Yes	0.28	0.07	0.003	0.85	0.006	0.14	-0.06
	(0.09 ; 0.67)	(-0.22 ; 0.36)	(-0.005 ; 0.012)	(0.06 ; 1.66)	(0.001 ; 0.011)	(0.02 ; 0.27)	(-0.50 ; 0.38)

[a] Adjusted for: Sex, family income at birth, maternal skin colour, maternal schooling, maternal age at childbirth, parity, pre-gestational nutritional status and maternal height

[b] Adjusted for: Sex, family income at birth, maternal skin colour, maternal schooling, maternal age at childbirth, parity, pre-gestational nutritional status, maternal height and partner smoking at 4 years

[c] Adjusted for: Sex, family income at birth, maternal skin colour, maternal schooling, maternal age at childbirth, parity, pre-gestational nutritional status, maternal height and maternal smoking in pregnancy

[d] Adjusted for: Sex, family income at birth, maternal skin colour, maternal schooling, maternal age at childbirth, parity, pre-gestational nutritional status, maternal height and partner smoking in pregnancy

For peer review only - http://bmjopen.bmj.com/site/about/guidelines.xhtml

	G-computation estimate (95%CI)

For peer review only

Table 5. Analysis of mediation in the association between maternal smoking during pregnancy and body composition measures at adulthood in the 1982 (at 30 years) and 1993 (22 years) Pelotas birth cohorts, Brazil.

For peer review only - http://bmjopen.bmj.com/site/about/guidelines.xhtml

**Anthropometric and body composition measures**	**Mediators**	**Natural direct effect**	**Natural indirect effect**	**Mediated effect (%)**
Body mass index (kg/m^2)	Birth weight	0.79 (0.44 ; 1.15)	0.13 (-0.05 ; 0.31)	14.1
	Weight gain from birth to two years	0.01 (-0.53 ; 0.54)	0.21 (-0.08 ; 0.50)	**96.2**
Fat mass index (kg/m^2)	Birth weight	0.44 (0.19 ; 0.70)	-0.03 (-0.16 ; 0.10)	7.7
	Weight gain from birth to two years	-0.02 (-0.40 ; 0.35)	-0.06 (-0.27 ; 0.14)	**71.7**
Android to gynoid fat ratio	Birth weight	0.014 (0.006 ; 0.022)	-0.002 (-0.006 ; 0.002)	17.1
	Weight gain from birth to two years	0.018 (0.005 ; 0.030)	0.001 (-0.006 ; 0.007)	3.4
Waist circumference (cm)	Birth weight	2.02 (1.28 ; 2.76)	0.18 (-0.22 ; 0.57)	8.1
	Weight gain from birth to two years	0.12 (-1.03 ; 1.26)	0.72 (0.09 ; 1.34)	**86.1**
Waist to height ratio	Birth weight	0.012 (0.007 ; 0.016)	0.001 (-0.002 ; 0.003)	3.0
	Weight gain from birth to two years	0.002 (-0.005 ; 0.009)	0.003 (-0.001 ; 0.007)	56.0
Lean mass index (kg/m^2)	Birth weight	0.37 (0.25 ; 0.48)	0.04 (-0.02 ; 0.10)	8.9
	Weight gain from birth to two years	0.16 (-0.02 ; 0.34)	-0.02 (-0.11 ; 0.08)	12.7
Height (cm)	Birth weight	-0.40 (-0.78 ; -0.02)	-0.18 (-0.39 ; 0.03)	30.9
	Weight gain from birth to two years	-0.93 (-1.52 ; -0.33)	0.10 (-0.24 ; 0.43)	11.5

For peer review only

For peer review only - http://bmjopen.bmj.com/site/about/guidelines.xhtml

STROBE 2007 (v4) Statement—Checklist of items that should be included in reports of *cohort studies*

Section/Topic	Item #	Recommendation	Reported on page #
**Title and abstract**	1	(*a*) Indicate the study's design with a commonly used term in the title or the abstract	1
		(*b*) Provide in the abstract an informative and balanced summary of what was done and what was found	2
**Introduction**			
Background/rationale	2	Explain the scientific background and rationale for the investigation being reported	4
Objectives	3	State specific objectives, including any prespecified hypotheses	5
**Methods**			
Study design	4	Present key elements of study design early in the paper	5
Setting	5	Describe the setting, locations, and relevant dates, including periods of recruitment, exposure, follow-up, and data collection	5 to 8
Participants	6	(*a*) Give the eligibility criteria, and the sources and methods of selection of participants. Describe methods of follow-up	5 to 8
		(*b*) For matched studies, give matching criteria and number of exposed and unexposed	Not applicable
Variables	7	Clearly define all outcomes, exposures, predictors, potential confounders, and effect modifiers. Give diagnostic criteria, if applicable	5 to 7
Data sources/ measurement	8*	For each variable of interest, give sources of data and details of methods of assessment (measurement). Describe comparability of assessment methods if there is more than one group	5 to 7
Bias	9	Describe any efforts to address potential sources of bias	5 to 7
Study size	10	Explain how the study size was arrived at	5 to 7
Quantitative variables	11	Explain how quantitative variables were handled in the analyses. If applicable, describe which groupings were chosen and why	5 to 7
Statistical methods	12	(*a*) Describe all statistical methods, including those used to control for confounding	7
		(*b*) Describe any methods used to examine subgroups and interactions	Not applicable
		(*c*) Explain how missing data were addressed	8
		(*d*) If applicable, explain how loss to follow-up was addressed	8
		(*e*) Describe any sensitivity analyses	Not applicable
**Results**			

For peer review only

Participants	13*	(a) Report numbers of individuals at each stage of study—eg numbers potentially eligible, examined for eligibility, confirmed eligible, included in the study, completing follow-up, and analysed	8
		(b) Give reasons for non-participation at each stage	8
		(c) Consider use of a flow diagram	8
Descriptive data	14*	(a) Give characteristics of study participants (eg demographic, clinical, social) and information on exposures and potential confounders	8 and 20
		(b) Indicate number of participants with missing data for each variable of interest	8 and 20
		(c) Summarise follow-up time (eg, average and total amount)	8 and 20
Outcome data	15*	Report numbers of outcome events or summary measures over time	8 and 20
Main results	16	(*a*) Give unadjusted estimates and, if applicable, confounder-adjusted estimates and their precision (eg, 95% confidence interval). Make clear which confounders were adjusted for and why they were included	8, 9 and 21 to 23
		(*b*) Report category boundaries when continuous variables were categorized	20
		(*c*) If relevant, consider translating estimates of relative risk into absolute risk for a meaningful time period	Not applicable
Other analyses	17	Report other analyses done—eg analyses of subgroups and interactions, and sensitivity analyses	Not applicable
**Discussion**			
Key results	18	Summarise key results with reference to study objectives	10
**Limitations**			
Interpretation	20	Give a cautious overall interpretation of results considering objectives, limitations, multiplicity of analyses, results from similar studies, and other relevant evidence	12
Generalisability	21	Discuss the generalisability (external validity) of the study results	12
**Other information**			
Funding	22	Give the source of funding and the role of the funders for the present study and, if applicable, for the original study on which the present article is based	13

*Give information separately for cases and controls in case-control studies and, if applicable, for exposed and unexposed groups in cohort and cross-sectional studies.

Note: An Explanation and Elaboration article discusses each checklist item and gives methodological background and published examples of transparent reporting. The STROBE checklist is best used in conjunction with this article (freely available on the Web sites of PLoS Medicine at http://www.plosmedicine.org/, Annals of Internal Medicine at http://www.annals.org/, and Epidemiology at http://www.epidem.com/). Information on the STROBE Initiative is available at www.strobe-statement.org.

For peer review only

BMJ Open

Maternal smoking during pregnancy and offspring body composition in adulthood: Results from two birth cohort studies

Journal:	*BMJ Open*
Manuscript ID	bmjopen-2018-023852.R1
Article Type:	Research
Date Submitted by the Author:	13-Jul-2018
Complete List of Authors:	Magalhães, Elma Izze; Universidade Federal de Pelotas, Programa de Pós-Graduação em Epidemiologia<br>Lima, Natália; Universidade Federal de Pelotas, Programa de Pós-Graduação em Epidemiologia<br>Menezes, Ana Maria ; Universidade Federal de Pelotas, Programa de Pós-Graduação em Epidemiologia<br>Goncalves, Helen; Universidade Federal de Pelotas, Programa de Pós-Graduação em Epidemiologia<br>Wehrmeister, Fernando; Universidade Federal de Pelotas, Programa de Pós-Graduação em Epidemiologia<br>Assuncao, M.C; Universidade Federal de Pelotas, Programa de Pós-Graduação em Epidemiologia<br>Horta, Bernardo; Federal University of Pelotas, Postgraduate Program in Epidemiology
**Primary Subject Heading**:	Epidemiology
Secondary Subject Heading:	Nutrition and metabolism, Public health
Keywords:	Maternal smoking, Pregnancy, Body Composition, Cohort study

SCHOLARONE™
Manuscripts

Maternal smoking during pregnancy and offspring body composition in adulthood: Results from two birth cohort studies

Elma Izze da Silva Magalhães[1] — elmaizzenutri@gmail.com

Natália Peixoto Lima[1] — natyplima@hotmail.com

Ana Maria Baptista Menezes[1] — anamene.epi@gmail.com

Helen Gonçalves[1] — hdgs.epi@gmail.com

Fernando C. Wehrmeister[1] — fcwehrmeister@gmail.com.br

Maria Cecília Formoso Assunção[1] — cecilia.epi@gmail.com

Bernardo Lessa Horta[1] — blhorta@gmail.com

[1]Postgraduate Programme in Epidemiology, Federal University of Pelotas, Pelotas, Rio Grande do Sul, Brazil.

Correspondence to:

Elma Izze da Silva Magalhães. Postgraduate Programme in Epidemiology, Rua Marechal Deodoro, 1160, 3º piso, Centro, Pelotas, RS, Brazil. Cep: 96020-220 - Caixa Postal 464. Telephone number: +55 53 98159-5411. E-mail address: elma_izze@hotmail.com.

Word count: 3,615

For peer review only - http://bmjopen.bmj.com/site/about/guidelines.xhtml

ABSTRACT

Objective: To evaluate the association of maternal smoking during pregnancy with offspring body composition in adulthood and explore the causality of this association.

Design: Birth cohort.

Setting: Population-based study in Pelotas, Brazil.

Participants: All newborn infants in the city's hospitals were enrolled in 1982 and 1993. At a mean age of 30.2 and 22.6 years, the 1982 and 1993 cohorts, respectively, followed the subjects and 7,222 subjects were evaluated.

Primary outcome measures: Body mass index, fat mass index, android to gynoid fat ratio, waist circumference, waist to height ratio, lean mass index and height.

Results: Prevalence of maternal smoking during pregnancy was 35.1% and 32.6%, in 1982 and 1993 cohorts, respectively. Offspring of smoking mothers showed higher mean body mass index (β: 0.84; 95%CI: 0.55 to 1.12 kg/m^2), fat mass index (β: 0.44; 95%CI: 0.23 to 0.64 kg/m^2), android to gynoid fat ratio (β: 0.016; 95%CI: 0.010 to 0.023), waist circumference (β: 1.74; 95%CI: 1.15 to 2.33 cm), waist to height ratio (β: 0.013; 95%CI: 0.010 to 0.017) and lean mass index (β: 0.33; 95%CI: 0.24 to 0.42 kg/m^2), whereas height was lower (β: -0.95; -1.26 to -0.65). Weight gain in the first two years captured most of the association of maternal smoking with body mass index (96.2%), waist circumference (86.1%) and fat mass index (71.7%).

Conclusions: Maternal smoking in pregnancy was associated with offspring body composition measures in adulthood.

Keywords: Maternal smoking; Pregnancy; Body Composition; Cohort study.

For peer review only - http://bmjopen.bmj.com/site/about/guidelines.xhtml

ARTICLE SUMMARY

Strengths and limitations of this study

- High follow-up rate, and the lack of differences in the follow-up rate according to maternal smoking during pregnancy, suggesting that selection bias is unlikely.
- We increased causal inference by comparing the estimates of maternal and paternal smoking.
- Body composition was assessed using dual X-ray absorptiometry and air-displacement pletysmography.
- We evaluated smoking in pregnancy using a questionnaire and the information was not validated with biochemical markers.
- Information on partner smoking was obtained at different times in the two cohorts.

For peer review only

INTRODUCTION

Maternal smoking during pregnancy has clear short-term consequences, increasing the risk of low birthweight and intrauterine growth retardation.[1] Moreover, maternal smoking is also associated with increased infant mortality rate, and cessation of smoking during pregnancy reduces the risk of infant death.[2] However, evidence on the long-term consequences of maternal smoking are not clear-cut. Meta-analysis by Brion et al.[3] related that blood pressure was slightly higher among offspring of smoking mothers. It has also been reported a higher risk of diabetes.[4] On the other hand, Horta et al.[5] observed that the association between maternal smoking in the pregnancy and metabolic cardiovascular risk factors in early adulthood was mediated by offspring lifestyle in adulthood.

With respect to body composition, it has been suggested that maternal smoking in pregnancy is positively associated with adiposity and risk of overweight/obesity in the offspring.[6, 7] On the other hand, because socioeconomic status is negatively associated with maternal smoking[8] and obesity,[9] the association of maternal smoking with offspring obesity could be due to residual confounding.[10] Indeed, some studies have reported that after controlling for socioeconomic and environmental variables, the magnitude of the association decreased.[11, 12]

Because socioeconomic status is usually assessed using few variables, the full dimension of socioeconomic status may not have been captured. And, residual confounding may be observed even among those studies that adjusted the estimates to socioeconomic and demographic variables. Comparison of the magnitude of the associations with maternal and paternal smoking is a strategy that can be used to increase causal inference. Similar associations for paternal and maternal smoking would suggest that confounding by

For peer review only - http://bmjopen.bmj.com/site/about/guidelines.xhtml

socioeconomic, environmental or familiar variables is an explanation for the observed associations. On the other hand, if the association is stronger for maternal variables, it is unlikely that the observed association is due to residual confounding.[13]

This study was aimed at assessing the association of maternal smoking during pregnancy with offspring body composition in adulthood and explore the causality of this association.

METHODS

Study Design and Population

This study is based on data from two birth cohorts carried out in Pelotas, a southern Brazilian city. In 1982 and 1993, in the perinatal study, all maternity hospitals in the city were visited daily, and the births identified. Those liveborns whose families lived in the urban area of the city were examined and their mothers interviewed soon after delivery. These subjects have been followed-up for several times through the life cycle, and further details on the study methodology have been published elsewhere.[14, 15]

Briefly, during the whole of 1982, among the births to mothers living in the urban area of Pelotas, 5,914 newborns and their mothers were enrolled e accepted to participate in the perinatal study. After, follow-up visits were carried at the ages of 1, 2, 4, 13, 15, 18, 19, 23 and 30 years. Most visits included subsamples of the cohort, except for those at 2, 4, 23 and 30 years, in which we attempted to locate the whole cohort. Between June 2012 and February 2013, 4,534 members were located, in which 467 were living far from Pelotas, 86 refused and another 280 did not attend the interview in spite of repeated invitations. Thus, a total of 3,701 subjects were interviewed at age 30 years, which adding the to the number of participants known to have died (n=325) made up 68.1% of the original cohort.

For peer review only - http://bmjopen.bmj.com/site/about/guidelines.xhtml

Similarly, among the births occurring in Pelotas during the year of 1993 (N=5,265), 5,249 subjects and your mothers were enrolled and agreed to take part in the longitudinal study. Subsamples of the cohort were evaluated in the follow-up visits were carried at the ages of 1, 3 and 6 months and at 1 and 4 years. In the follow-up visits at 11, 15, 18 and 22 years the whole cohort was recruited. The follow-up at 22 years occurred October 2015 to July 2016, and of the original cohort, 4933 were found. Among the located members, 175 refused to participate and 1071 were considered losses. Those who were interviewed (N=3,810), added to those known to have died (N=193), represent a retention rate of 76.3%. The follow-up rates at each follow-up visit of the 1982 and 1993 cohort are described in Supplementary Table 1.

Exposure Variables

In both cohorts, information on maternal smoking during pregnancy, duration and numbers of cigarettes smoked per day were obtained retrospectively from the mothers shortly after delivery, in the perinatal study. Those mothers who reported any tobacco use during pregnancy were considered as smokers, and they were asked whether they had smoked during the whole pregnancy and how many cigarettes on average they had smoked per day. In addition, we also collected information on partner smoking. In the 1993 cohort, this information was gathered based on the mothers report, in the perinatal study, whereas in the 1982 cohort, information on partner smoking was provided by the mother in the follow-up visit at 4 years of age.

Outcome Variables

For peer review only - http://bmjopen.bmj.com/site/about/guidelines.xhtml

In follow-up at 30 and 22 years, subjects were invited to visit the research clinic to be interviewed, examined and donate a blood sample. Weight was measured using the Bod Pod scale and height with a portable stadiometer (accuracy of 0.1 cm). Body mass index (BMI) was estimated by dividing weight (in kg) by square height (in meters).

Waist circumference (in cm) was measured with the subject standing, with the arms hanging freely and next to the body, using a non-elastic measuring tape in the horizontal plane around the narrowest part of the waist. In obese subjects, the measure was taken in the horizontal plane at the point between the last rib and the iliac crest. Waist to height ratio was estimated by dividing waist circumference by height (both in cm).

Fat-free and fat mass (total and in the android and gynoid regions) were assessed using dual-energy x-ray absorptiometry (DXA). Lean and fat mass indexes were estimated by dividing fat-free and fat mass (in kg) by square height in meters. Android to gynoid fat ratio was estimated by dividing percentage fat in region android by percentage fat in gynoid region. Pregnant women or those in the postpartum period (three months) were excluded from this assessment.

Confounders

The following variables were considered as possible confounders: sex, age in the assessment of the outcomes, family income at birth (total income in the month before the interview), maternal skin color (self-reported), maternal schooling (years of schooling successfully completed), maternal age at childbirth, parity (number of previous deliveries) and pre-gestational nutritional status. Information about sex of the child, family income at birth , maternal skin color, maternal schooling, maternal age at childbirth and parity were collected through a questionnaire applied to the mothers in the interview of the perinatal study. To

For peer review only - http://bmjopen.bmj.com/site/about/guidelines.xhtml

evaluate the pre-gestational nutritional status, information on prepregnancy maternal weight was obtained from the antenatal care records or by maternal recall and height was measured by the research team in the interview immediately after delivery. The exact age in the assessment of outcomes was reported by subjects in the 30 and 22 years follow-up in the 1982 and 1993 cohorts, respectively.

Mediators

Birthweight and weight gain from birth to two years, were considered as possible mediators. Birthweight (in grams) was assessed soon after delivery, by the hospital staff, using paediatric scales that were calibrated weekly by the research team. Weight gain from birth to two years of age was obtained by the difference in z-score between the child's weight at 2 years of age and the birthweight. The weight of the child at two years of age was measured only in the follow-up of the 1982 cohort. Thus, this analysis was only performed only for the 1982 cohort.

Statistical Analysis

Stata, version 14.0 (StataCorp, College Station, TX) was used in the analysis. Data from the two cohorts were analysed together, because both cohorts showed similar association between maternal smoking and offspring anthropometry (data not shown). Means were compared using Student t-test and analysis of variance (ANOVA). Multiple linear regression was used to obtain estimates that were adjusted for confounding variables. G-computation (stata package: st0238) was used to estimate direct and indirect effects of maternal smoking in pregnancy on offspring body composition in adulthood (bootstrap replications: 10,000). In the analysis for birthweight, gestational age was considered as post-confounder (confounders in

For peer review only

the relationship between the mediator and the outcome), while for weight gain from birth to two years, we considered family income in childhood as a post-confounder. Sex, age at body composition assessment, family income at birth, parity, pre-gestational BMI, skin colour, maternal schooling, and height were included as base-confounders.

Patient and Public Involvement

The study was approved by the Ethical Review Board of the Faculty of Medicine of the Federal University of Pelotas, and was performed in accordance with the ethical standards established in the 1964 Declaration of Helsinki and its later amendments. Written informed consent was obtained from participating subjects.

RESULTS

Among the 3,701 and 3,810 participants of the 1982 and 1993 cohort on follow-up at 30 and 22 years, respectively, information on maternal smoking and at least one of the outcomes were available for 3,626 participants from 1982 cohort and 3,596 from 1993. Analysing the follow-up rates in the last visit of each cohort, according to baseline characteristics, was observed that in the 1982 the cohort follow-up rates at 30 years were slightly higher among females, those who were born preterm and those in the intermediate socioeconomic categories. In the 1993 cohort, losses to follow-up were higher among males and at the extremes of the income distribution. Nevertheless, follow-up rates among different subgroups are reasonably similar, ranging from 60% to 75% in all variables studied, so that attrition bias is unlikely (Supplementary Table 2).

Table 1 shows the characteristics of the subjects included in the analyses. The proportion of preterm births was 5.6% and 8.0% in 1982 and 1993, respectively. The

For peer review only

prevalence of overweight/obesity was 43.2% at 22 years (1993 cohort) and 57.6% at 30 years (1982 cohort). Prevalence maternal smoking during pregnancy was 35.0% and 32.4% in 1982 and 1993, respectively. In the 1993 cohort, 32.4% of the partners smoked during pregnancy, whereas, in the 1982 cohort, 58.8% of the partners were smoker at the follow-up visit at 4 years of age.

Supplementary Table 3 shows that prevalence of maternal smoking during whole pregnancy was significantly higher among the mothers in the lower income tertile and lower schooling in both cohorts. On the order hand, maternal smoking during pregnancy was associated only with the partner smoking in pregnancy, in the 1993 cohort, but no with the partner smoking at 4 years, in the 1982 cohort.

In the crude analyses (Table 2), maternal smoking in the pregnancy was associated with higher mean BMI, fat mass index, android to gynoid fat ratio, waist circumference, waist to height ratio, as well as, lean mass index ($P<0.05$). On the other hand, offspring of mothers who smoked during pregnancy had lower height ($P<0.0001$). With respect to duration of maternal smoking during pregnancy, body mass, fat mass index, android to gynoid fat ratio, waist circumference, waist to height ratio and lean mass index were slightly higher among the offspring of mothers who smoked only in part of pregnancy in relation to those whose mother smoked in the whole pregnancy. But the lower means were observed among those subjects whose mothers did not smoke in the pregnancy ($P<0.05$). On the other hand, height was inversely associated with the duration of maternal smoking during pregnancy ($P<0.0001$). Regarding partner, smoking, no significant differences were observed for partner smoking at 4 years (cohort 1982). On the other hand, lower means of waist circumference, waist to height ratio, lean mass index and height were observed in the offspring in which the partners smoked during pregnancy ($P <0.05$).

For peer review only - http://bmjopen.bmj.com/site/about/guidelines.xhtml

After controlling for confounding variables, offspring of smoking mothers showed higher body mass index, fat mass index, android to gynoid fat ratio, waist circumference, waist to height ratio and lean mass index, whereas height was lower. Concerning, the duration and intensity of maternal smoking during pregnancy, we only observed a dose response association for height, whereas for the remaining body composition outcomes, the regression coefficients tend to slightly higher among those subjects whose mother stopped smoking in the pregnancy or smoked from 1 to 14 cigarettes/day. (Table 3). Although not be objective of the study, we also tested the association between maternal smoking in gestation and overweight in the offspring, and found a positive association even after adjusting for confounding factors (OR Adjusted: 1.41; 95%CI: 1.26 to 1.57).

Table 4 shows that maternal smoking was associated with most of the body composition outcomes, even after controlling for partner smoking. Moreover, in both cohorts the magnitude of the associations were stronger for maternal than partner smoking. Because information on partner smoking during pregnancy was not available for the 1982 cohort, we used the information on partner smoking collected in the follow-up visit at 4 years of age.

Concerning the mediation analysis, birthweight captured a small portion of the associations, whereas weight gain in the first two years captured most of the association of maternal smoking with body mass index (96.2%), fat mass index (71.7%) and waist circumference (86.1) - Table 5.

DISCUSSION

Our findings suggest that exposure to maternal tobacco smoking in utero increases adiposity in early adulthood. These associations were observed even after controlling for several confounding variables. Furthermore, we observed that the magnitude of the

For peer review only - http://bmjopen.bmj.com/site/about/guidelines.xhtml

association was higher for maternal than paternal smoking and even after controlling for paternal smoking, maternal smoking during pregnancy was associated with body composition in adulthood. Suggesting, therefore, that residual confounding is an unlikely explanation for the association between maternal smoking and offspring body composition.

The results of our study corroborate the findings from previous studies that have observed a positive association between maternal smoking during pregnancy and offspring adiposity.[11, 12, 16] In relation to central adiposity, it has also been reported higher means waist circumference[16] and android to gynoid fat ratio.[17] Such measures are indicators of fat accumulation in the abdominal region and are more related to cardiovascular risk.[18]

Concerning the mechanisms underlying the association between maternal smoking during pregnancy and offspring adiposity, it has been suggested that this association is due to the nicotine, present in tobacco. In both humans and animals, nicotine when crossing the placenta acts centrally and peripherally as a suppressant of appetite and body weight, resulting in hyperphagia and weight gain when the offspring is no longer exposed to nicotine in postnatal period.[19, 20] In addition, exposure to nicotine in gestation may result in increase in body adiposity through alterations in endocrine control of body weight homeostasis.[21] In animals, prenatal exposure to nicotine decreased responsiveness to adrenergic stimuli and promoted rapid weight gain.[22] Similarly, prenatal exposure to nicotine in humans may decrease responsiveness to adrenergic stimuli by epinephrine and norepinephrine, which modulate the mobilization of lipids from adipose tissue.[23]

It has been reported that low birthweight is associated with increased risk of obesity later in life,[24, 25] but in the present study we did not find any evidence that birthweight mediated the observed associations. On the other hand, weight gain in early childhood captured an important proportion of the association of maternal smoking in pregnancy with

For peer review only

BMI, fat mass index and waist circumference. Maternal smoking tends to decrease foetal growth, which would be compensated by rapid postnatal weight gain.[26] Because offspring of smoking mothers were shorter, it seems that weight gain was higher than linear growth. Indeed, Adair et al.[27] reported that faster relative weight gain in the first two years was associated with a higher risk of overweight/obesity in adulthood.

In our study, we observed that lean mass was higher among offspring of mothers who smoked in pregnancy. Leary et al.[11] also observed that lean mass was higher among subjects whose mother smoked in the pregnancy. These authors suggested that this association with lean mass would be simply due to the association with total body mass. Indeed, subjects with higher total body mass would have higher fat and lean mass. Concerning the association with height, a negative association has already been reported by previous studies.[12, 28]

Our study has many strengths, the data was collected prospectively and information on maternal smoking during pregnancy was collected soon after delivery, with a short recall time. Previous studies on this subject are mainly retrospective, or cross-sectional,[28, 29] and data on maternal smoking was gathered with a long recall time, which can lead to misclassification. Secondly, the high follow-up rates, even after 30 years and the lack of differences in the follow-up rate according to maternal smoking during pregnancy suggests that selection bias is unlikely.[14] Finally, most of the previous studies have evaluated only BMI as a measure of adiposity. We used several anthropometric and body composition measurements, including fat and lean mass measured using DXA. By comparing the estimates of maternal smoking to partner smoking, we increased causal inference and the analysis suggested that the observed associations were unlikely to be due to residual confounding.

With respect to the limitations, information on parental smoking was gathered using a questionnaire and the information was not validated with biochemical markers. Because

For peer review only - http://bmjopen.bmj.com/site/about/guidelines.xhtml

smoking in pregnancy is a socially disapproved act, some mothers may have occulted the information on prenatal smoking,[30] underestimating the prevalence of smoking during pregnancy. Because this classification error was independent of body composition, which was measured many years later, the misclassification would be non-differential. Because non-differential misclassification tends to underestimate the magnitude of the associations, the observed associations are not due to such error. Another limitation is the fact that information on partner smoking was obtained at different times in the two cohorts. However, it is likely that parents who smoked during pregnancy remained smokers after birth and the first years of the offspring's life. In addition, no discrepant results were observed regarding the effect of paternal smoking among the two cohorts.

In conclusion, we observed an association between maternal smoking in pregnancy and body composition measures in adulthood. The specificity of the association for maternal, in relation to paternal smoking, suggested that this association is not due to residual confounding. Analysis of mediation suggest the weight gain from birth to two years is an important mediator of the association between maternal smoking during pregnancy and adiposity in adulthood.

CONTRIBUTORSHIP STATEMENT

EISM designed the study, performed the statistical analysis, interpretation of the results and drafted the manuscript. BLH and NPL designed the study, helped the data analysis, and participated in the preparation of the manuscript. BLH coordinated the follow-up of the 1982 cohort and AMBM coordinated the follow-up of the 1993 cohort. AMBM, HDG, FCW, and MCA helped in the data acquisition and interpretation of the data. All authors revised and approved the final version of the manuscript. Each author contributed

For peer review only

important intellectual content during manuscript drafting or revision and accepts accountability for the overall work by ensuring that questions pertaining to the accuracy or integrity of any portion of the work are appropriately investigated and resolved.

COMPETING INTERESTS

None declared.

FUNDING

This article is based on data from the study "Pelotas Birth Cohort, 1982 and 1993" conducted by the Postgraduate Program in Epidemiology at Federal University of Pelotas with the collaboration of the Brazilian Public Health Association (ABRASCO). From 2004 to 2016, the Wellcome Trust (086974/Z/08/Z) supported the Pelotas birth cohort study. The International Development Research Center, World Health Organization, Overseas Development Administration, European Union, National Support Program for Centers of Excellence (PRONEX), the Brazilian National Research Council (CNPq), and the Brazilian Ministry of Health supported previous phases of the study.

DATA SHARING STATEMENT

Data are available through the Centre of Epidemiological Research and the Postgraduate Program in Epidemiology.

ACKNOWLEDGEMENTS

We acknowledge the contributions of 1982 and 1993 Pelotas Cohorts participants, others researchers and staff.

For peer review only

REFERENCES

1. Kramer MS. Determinants of low birthweight: methodological assessment and meta-analysis. *Bull World Health Organ* 1987;65:663–737.

2. Johansson ALV, Dickman PW, Kramer MS, et al. Maternal smoking and infant mortality: does quitting smoking reduce the risk of infant death?. *Epidemiology* 2009;20:590–597.

3. Brion MJA, Leary SD, Lawlor DA, et al AR. Modifiable maternal exposures and offspring blood pressure: a review of epidemiological studies of maternal age, diet, and smoking. *Pediatr Res* 2008; 63:593–598.

4. Montgomery SM, Ekbom A. Smoking during pregnancy and diabetes mellitus in a British longitudinal birth cohort. *BMJ* 2002;324:26–27.

5. Horta BL, Gigante DP, Nazmi A, et al. Maternal smoking during pregnancy and risk factors for cardiovascular disease in adulthood. *Atherosclerosis* 2011;219:815–820.

6. Oken E, Levitan EB, Gillman MW. Maternal smoking during pregnancy and child overweight: systematic review and meta-analysis. *Int J Obes* 2008; 32:201–210.

For peer review only

For peer review only - http://bmjopen.bmj.com/site/about/guidelines.xhtml

7. Ino T. Maternal smoking during pregnancy and offspring obesity: Meta-analysis. *Pediatr Int* 2010; 52:94–99.

8. Iliadou AN, Koupil I, Villamor E, Altman D, Hultman C, Langström N, et al. Familial factors confound the association between maternal smoking during pregnancy and young adult offspring overweight. *Int J Epidemiol* 2010;39:1193–1202.

9. Monteiro CA, Moura EC, Conde WL, et al. Socioeconomic status and obesity in adult populations of developing countries: a review. *Bull World Health Organ* 2004;82:940–946.

10. Riedel C, Schönberger K, Yang S, et al. Parental smoking and childhood obesity: higher effect estimates for maternal smoking in pregnancy compared with paternal smoking—a meta-analysis. *Int J Epidemiol* 2014;43:1593–1606.

11. Leary SD, Smith GD, Rogers IS, et al. Smoking during pregnancy and offspring fat and lean mass in childhood. *Obesity* 2006;14:2284–2293.

12. Chen A, Pennell ML, Klebanoff MA, et al. Maternal smoking during pregnancy in relation to children overweight: Follow-up to age 8 years. *Int J Epidemiol* 2006;35:121–130.

13. Howe LD, Matijasevich A, Tilling K, et al. Maternal smoking during pregnancy and offspring trajectories of height and adiposity: comparing maternal and paternal associations. *Int J Epidemiol* 2012;41:722–732.

For peer review only

For peer review only - http://bmjopen.bmj.com/site/about/guidelines.xhtml

14. Horta BL, Gigante DP, Gonçalves H, et al. Cohort profile update: the 1982 Pelotas (Brazil) birth cohort study. *Int J Epidemiol* 2015;44:441–441e.

15. Gonçalves H, Wehrmeister FC, Assunção MC, et al. Cohort Profile Update: The 1993 Pelotas (Brazil) Birth Cohort follow-up at 22 years. *Int J Epidemiol* 2017;1–7.

16. Mamun AA, O'Callaghan MJ, Williams GM, et al. Maternal smoking during pregnancy predicts adult offspring cardiovascular risk factors-evidence from a community-based large birth cohort study. *PloS One* 2012;7:e41106.

17. Durmuş B, Heppe DHM, Taal HR, et al. Parental smoking during pregnancy and total and abdominal fat distribution in school-age children: the Generation R Study. *Int J Ob* 2014;38:966–972.

18. Lee CMY, Huxley RR, Wildman RP, et al. Indices of abdominal obesity are better discriminators of cardiovascular risk factors than BMI: a meta-analysis. *J Clin Epidemiol* 2008;61:646–653.

19. Li MD, Parker SL, Kane JK. Regulation of feeding-associated peptides and receptors by nicotine. *Mol Neurobiol* 2000;22:143–165.

20. Jo YH, Talmage DA, Role LW. Nicotinic receptor□mediated effects on appetite and food intake. *J Neurobiol* 2002;53:618–632.

For peer review only

For peer review only - http://bmjopen.bmj.com/site/about/guidelines.xhtml

21. Bruin JE, Gerstein HC, Holloway AC. Long-term consequences of fetal and neonatal nicotine exposure: a critical review. *Toxicol Sci* 2010;116:364–374.

22. Levin ED. Fetal nicotinic overload, blunted sympathetic responsivity, and obesity. *Birth Defects Res A Clin Mol Teratol* 2005;73:481–484.

23. Schiffelers SLH, Saris WHM, Boomsma F, et al. β1-and β 2-Adrenoceptor-Mediated Thermogenesis and Lipid Utilization in Obese and Lean Men. *J Clin Endocrinol Metab* 2001;86:2191–2199.

24. Malina, RM, Katzmarzyk PT, Beunen G. Birthweight and its relationship to size attained and relative fat distribution at 7 to 12 years of age. *Obesity* 1996; 4:385–390.

25. Singhal A, Wells J, Cole TJ, et al. Programming of lean body mass: a link between birthweight, obesity, and cardiovascular disease?. *Am J Clin Nutr* 2003;77:726–730.

26. Oken E, Gillman MW. Fetal origins of obesity. *Obesity* 2003;11:496–506.

27. Adair LS, Fall CH, Osmond C, et al. Associations of linear growth and relative weight gain during early life with adult health and human capital in countries of low and middle income: findings from five birth cohort studies. *Lancet* 2013;382:525–534.

For peer review only

For peer review only - http://bmjopen.bmj.com/site/about/guidelines.xhtml

28. Koshy G, Delpisheh A, Brabin BJ. Dose response association of pregnancy cigarette smoke exposure, childhood stature, overweight and obesity. *Eur J Public Health* 2011; 21:286–291.

29. Adams AK, Harvey HE, Prince R.J. Association of maternal smoking with overweight at age 3 y in American Indian children. *Am J Clin Nutr* 2005; 82:393–398.

30. Dietz PM, Homa D, England LJ, et al. Estimates of nondisclosure of cigarette smoking among pregnant and nonpregnant women of reproductive age in the United States. *Am J Epidemiol* 2011; 173:355–359.

For peer review only

For peer review only - http://bmjopen.bmj.com/site/about/guidelines.xhtml

Table 1. Characteristics of population included in the present study, Pelotas, Brazil.

	1982 Cohort (N= 3,626)	1993 Cohort (N= 3,596)

For peer review only - http://bmjopen.bmj.com/site/about/guidelines.xhtml

Variable	N (%) [a]	Mean (SD) [b]	N (%) [a]	Mean (SD) [b]
**Sex**				
Male	1,766 (48.7)		1,706 (47.4)	
Female	1,860 (51.3)		1,890 (52.6)	
**Gestational age (weeks)**				
< 37	164 (5.6)		283 (8.0)	
37 to 39	1,336 (45.8)		2,741 (77.2)	
≥ 40	1,420 (48.6)		527 (14.8)	
**Birth weight (g)**				
< 2,500	280 (7.7)		338 (9.4)	
2,500 a 2,999	956 (26.4)		942 (26.2)	
≥ 3,000	2,389 (65.9)		2,314 (64.4)	
**Skin colour**				
White	2,750 (75.8)		2,163 (63.5)	
Non-white	876 (24.2)		1,245 (36.5)	
**Maternal smoking during pregnancy**				
Non smokers	2,353 (65.0)		2,430 (67.6)	
Smoked in part of pregnancy	276 (7.6)		160 (4.4)	
Smoked in whole pregnancy	990 (27.4)		1,006 (28.0)	
**Partner smoking** [c]				
No	1,166 (41.2)		2,433 (67.6)	
Yes	1,665 (58.8)		1,163 (32.4)	
**Familiar income at birth (minimum wage)**	715 (19.8)		628 (17.8)	
≤ 1	1,780 (49.3)		1,472 (41.7)	
1.1 to 3.0	706 (19.6)		877 (24.9)	
3.1 to 6.0	408 (11.3)		550 (15.6)	
> 6.0				
**Maternal schooling (years)**				
0 to 4	2,719 (75.1)		950 (26.5)	
5 to 8	399 (11.0)		1,695 (47.2)	
≥ 9	503 (13.9)		946 (26.3)	
**Body mass index in adulthood (kg/m^2)**		26.8 (5.5)		25.2 (5.3)
< 18.5	71 (2.0)		168 (4.7)	
18.5 to 24.9	1,426 (40.4)		1,855 (52.1)	
25.0 to 29.9	1,224 (34.7)		959 (27.0)	
≥ 30.0	810 (22.9)		577 (16.2)	
**Fat mass index (kg/m^2)**		8.7 (4.3)		7.9 (4.5)
**Android to gynoid fat ratio**		0.50 (0.14)		0.42 (0.12)
**Waist circumference (cm)**		84.8 (12.6)		80.0 (11.6)
**Waist to height ratio**		0.51 (0.07)		0.48 (0.07)
**Lean mass index (kg/m^2)**		16.7 (2.7)		16.1 (2.6)
**Height (cm)**		167.7 (9.2)		167.4 (9.5)

Abbreviation: SD, standard deviation; IQI, inter quartiles interval.
[a] For the categorical variables; [b] For the continuous variables; [c] At 4 years for the 1982 cohort and in pregnancy for the 1993 cohort.

For peer review only - http://bmjopen.bmj.com/site/about/guidelines.xhtml

Table 2. Mean for anthropometric and body composition measures in adulthood and according to maternal smoking during pregnancy and partner smoking.

	Mean (Standard deviation)						
	**Body mass index (kg/m²)**	**Fat mass index (kg/m²)**	**Android to gynoid fat ratio**	**Waist circumference (cm)**	**Waist to height ratio**	**Lean mass index (kg/m²)**	**Height (cm)**
**Maternal smoking in pregnancy** [a]	*P=<0.0001*****	*P=0.0090***	*P=<0.0001*****	*P=0.0001*****	*P=0.0001*****	*P=0.0001*****	*P=0.0001*****
No	25.8 (5.4)	8.2 (4.3)	0.46 (0.13)	82.0 (12.3)	0.49 (0.07)	16.3 (2.6)	168.1 (9.4)
Yes	26.4 (5.6)	8.5 (4.6)	0.47 (0.14)	83.2 (12.5)	0.50 (0.07)	16.6 (2.6)	166.6 (9.3)
**Duration of maternal smoking** [b]	*P=<0.0001*****	*P=0.0036***	*P=<0.0001*****	*P=<0.0001*****	*P=<0.0001*****	*P=<0.0001*****	*P=<0.0001*****
Non smokers	25.8 (5.4)	8.2 (4.3)	0.46 (0.13)	82.0 (12.3)	0.49 (0.07)	16.3 (2.6)	168.1 (9.4)
Smoked in part of pregnancy	27.0 (5.6)	9.0 (4.6)	0.48 (0.13)	84.7 (12.5)	0.50 (0.07)	16.7 (2.6)	167.8 (9.6)
Smoked in whole pregnancy	26.3 (5.6)	8.4 (4.6)	0.47 (0.14)	82.9 (12.4)	0.50 (0.07)	16.6 (2.6)	166.3 (9.2)
**Cigarettes smoked per day** [b]	*P=0.0001*****	*P=0.0165**	*P=<0.0001*****	*P=0.0007* [b]	*P=<0.0001*****	*P=<0.0001*****	*P=<0.0001*****
None	25.8 (5.4)	8.2 (4.3)	0.46 (0.13)	82.0 (12.3)	0.49 (0.07)	16.3 (2.6)	168.1 (9.4)
1 to 14 cigarettes/day	26.5 (5.6)	8.6 (4.6)	0.47 (0.14)	83.2 (12.5)	0.50 (0.07)	16.6 (2.6)	166.7 9.3)
≥ 15 cigarettes/day	26.3 (5.4)	8.3 (4.5)	0.48 (0.14)	83.2 (12.5)	0.50 (0.07)	16.7 (2.5)	165.9 (9.4)
**1982 cohort**							
**Partner smoking at 4 years** [a]	*P=0.4287*	*P=0.9976*	*P=0.3031*	*P=0.3249*	*P=0.5670*	*P=0.3963*	*P=0.1918*
No	26.8 (5.5)	8.4 (4.4)	0.50 (0.14)	84.9 (12.6)	0.51 (0.07)	16.7 (2.6)	167.9 (9.1)
Yes	26.7 (5.4)	8.4 (4.3)	0.49 (0.14)	84.4 (12.4)	0.50 (0.07)	16.6 (2.7)	167.4 (9.3)
**1993 cohort**							
**Partner smoking in pregnancy** [a]	*P=0.0794*	*P=0.8248*	*P=0.0816*	*P=0.0242**	*P=0.0004***	*P=0.0049***	*P=0.0099***
No	25.1 (5.1)	7.9 (4.3)	0.42 (0.11)	79.5 (11.3)	0.47 (0.06)	16.0 (2.5)	167.8 (9.4)
Yes	25.4 (5.6)	7.9 (4.7)	0.42 (0.12)	80.5 (12.1)	0.48 (0.07)	16.3 (2.6)	167.0 (9.6)

[a] Student t-test
[b] Analysis of variance (ANOVA)
*P<0.05; **P<0.01; ****P<0.0001.

For peer review only

For peer review only - http://bmjopen.bmj.com/site/about/guidelines.xhtml

Table 3. Adjusted regression coefficients for anthropometric and body composition measures in adulthood according to maternal smoking during pregnancy.

	Adjusted regression coefficient (95% confidence interval) [a]						
	**Body mass index (kg/m^2)**	**Fat mass index (kg/m^2)**	**Android to gynoid fat ratio**	**Waist circumference (cm)**	**Waist to height ratio**	**Lean mass index (kg/m^2)**	**Height (cm)**
**Maternal smoking in pregnancy**							
No	Reference	Reference	Reference	Reference	Reference	Reference	Reference
Yes	0.84 (0.55 ; 1.12)	0.44 (0.23 ; 0.64)	0.016 (0.010 ; 0.023)	1.74 (1.15 ; 2.33)	0.013 (0.010 ; 0.017)	0.33 (0.24 ; 0.42)	-0.95 (-1.26 ; -0.65)
**Duration of maternal smoking**							
Non-smokers	Reference	Reference	Reference	Reference	Reference	Reference	Reference
Smoked in part of pregnancy	1.17 (0.62 ; 1.71)	0.70 (0.30 ; 1.11)	0.013 (0.001 ; 0.026)	2.49 (1.32 ; 3.65)	0.016 (0.009 ; 0.023)	0.34 (0.16 ; 0.53)	-0.43 (-1.03 ; 0.18)
Smoked in whole Pregnancy	0.76 (0.46 ; 1.06)	0.37 (0.15 ; 0.59)	0.017 (0.010 ; 0.024)	1.56 (0.93 ; 2.19)	0.013 (0.009 ; 0.016)	0.32 (0.22 ; 0.42)	-1.07 (-1.40 ; -0.74)
**Cigarettes smoked per day**							
None	Reference	Reference	Reference	Reference	Reference	Reference	Reference
1 to 14 cigarettes/day	0.89 (0.60 ; 1.19)	0.46 (0.24 ; 0.69)	0.018 (0.011 ; 0.025)	1.86 (1.23 ; 2.50)	0.014 (0.010 ; 0.017)	0.34 (0.24 ; 0.44)	-0.81 (-1.15 ; -0.49)
≥ 15 cigarettes/day	0.62 (0.09 ; 1.15)	0.32 (-0.08 ; 0.71)	0.010 (-0.003 ;0.022)	1.24 (0.12 ; 2.36)	0.012 (0.005 ; 0.019)	0.30 (0.12 ; 0.47)	-1.52 (-2.11 ; -0.94)

[a] Adjusted for: Sex, age (Cohort), family income at birth, maternal skin colour, maternal schooling, maternal age at childbirth, parity, pre-gestational nutritional status and maternal height;

Table 4. Adjusted regression coefficients for anthropometric and body composition measures in adulthood according to maternal smoking during pregnancy and partner smoking.

For peer review only - http://bmjopen.bmj.com/site/about/guidelines.xhtml

	Adjusted regression coefficient (95% confidence interval)						
	**Body mass index (kg/m^2)**	**Fat mass index (kg/m^2)**	**Android to gynoid fat ratio**	**Waist circumference (cm)**	**Waist to height ratio**	**Lean mass index (kg/m^2)**	**Height (cm)**
**1982 cohort**							
**Maternal smoking in pregnancy [a]**							
No	Reference	Reference	Reference	Reference	Reference	Reference	Reference
Yes	0.65	0.30	0.020	1.54	0.012	0.27	-0.75
	(0.24 ; 1.06)	(0.01 ; 0.59)	(0.011 ; 0.030)	(0.67 ; 2.41)	(0.006 ; 0.017)	(0.13 ; 0.41)	(-1.17 ; -0.33)
**Partner smoking at 4 years [a]**							
No	Reference	Reference	Reference	Reference	Reference	Reference	Reference
Yes	-0.04	0.01	0.001	-0.05	0.001	0.02	-0.11
	(-0.47 ; 0.39)	(-0.29 ; 0.33)	(-0.010 ; 0.011)	(-0.97 ; 0.88)	(-0.005 ; 0.006)	(-0.12 ; 0.17)	(-0.56 ; 0.34)
**Maternal smoking in pregnancy [b]**							
No	Reference	Reference	Reference	Reference	Reference	Reference	Reference
Yes	0.64	0.24	0.016	1.60	0.012	0.24	-0.73
	(0.19 ; 1.10)	(-0.09 ; 0.56)	(0.005 ;0.027)	(0.62 ; 2.57)	(0.006 ; 0.018)	(0.09 ; 0.39)	(-1.21 ; -0.26)
**Partner smoking at 4 years [c]**							
No	Reference	Reference	Reference	Reference	Reference	Reference	Reference
Yes	-0.04	0.02	0.001	-0.05	0.001	0.02	-0.11
	(-0.47 ; 0.39)	(-0.29 ; 0.33)	(-0.010 ; 0.011)	(-0.97 ; 0.87)	(-0.005 ; 0.006)	(-0.12 ; 0.17)	(-0.55 ; 0.35)
**1993 cohort**							
**Maternal smoking in pregnancy [a]**							
No	Reference	Reference	Reference	Reference	Reference	Reference	Reference
Yes	1.04	0.38	0.014	1.99	0.016	0.38	-1.21
	(0.66 ; 1.42)	(0.25 ; 0.51)	(0.006 ; 0.023)	(1.18 ; 2.80)	(0.011 ; 0.021)	(0.25 ; 0.51)	(-1.66 ; -0.76)
**Partner smoking in pregnancy [a]**							
No	Reference	Reference	Reference	Reference	Reference	Reference	Reference
Yes	0.48	0.20	0.006	1.23	0.009	0.21	-0.28
	(0.11 ; 0.85)	(-0.09 ; 0.48)	(-0.002 ; 0.014)	(0.44 ; 2.01)	(0.004 ; 0.013)	(0.09 ; 0.34)	(-0.71 ; 0.15)
**Maternal smoking in pregnancy [d]**							
No	Reference	Reference	Reference	Reference	Reference	Reference	Reference
Yes	1.01	0.36	0.015	1.92	0.015	0.36	-1.13

For peer review only - http://bmjopen.bmj.com/site/about/guidelines.xhtml

Table 5. Analysis of mediation in the association between maternal smoking during pregnancy and body composition measures at adulthood in the 1982 (at 30 years) and 1993 (22 years) Pelotas birth cohorts, Brazil.

	(0.61 ; 1.42)	(0.23 ; 0.50)	(0.006 ;0.024)	(1.05 ; 2.78)	(0.009 ; 0.020)	(0.23 ; 0.50)	(-1.61 ; -0.65)
**Partner smoking in pregnancy** [c]							
No	Reference	Reference	Reference	Reference	Reference	Reference	Reference
Yes	0.28	0.07	0.003	0.85	0.006	0.14	-0.06
	(0.09 ; 0.67)	(-0.22 ; 0.36)	(-0.005 ; 0.012)	(0.06 ; 1.66)	(0.001 ; 0.011)	(0.02 ; 0.27)	(-0.50 ; 0.38)

[a] Adjusted for: Sex, family income at birth, maternal skin colour, maternal schooling, maternal age at childbirth, parity, pre-gestational nutritional status and maternal height
[b] Adjusted for: Sex, family income at birth, maternal skin colour, maternal schooling, maternal age at childbirth, parity, pre-gestational nutritional status, maternal height and partner smoking at 4 years
[c] Adjusted for: Sex, family income at birth, maternal skin colour, maternal schooling, maternal age at childbirth, parity, pre-gestational nutritional status, maternal height and maternal smoking in pregnancy
[d] Adjusted for: Sex, family income at birth, maternal skin colour, maternal schooling, maternal age at childbirth, parity, pre-gestational nutritional status, maternal height and partner smoking in pregnancy

For peer review only - http://bmjopen.bmj.com/site/about/guidelines.xhtml

Anthropometric and body composition measures	Mediators	G-computation estimate (95%CI)		Mediated effect (%)
		Natural direct effect	Natural indirect effect	
Body mass index (kg/m^2)	Birth weight	0.79 (0.44 ; 1.15)	0.13 (-0.05 ; 0.31)	14.1
	Weight gain from birth to two years	0.01 (-0.53 ; 0.54)	0.21 (-0.08 ; 0.50)	**96.2**
Fat mass index (kg/m^2)	Birth weight	0.44 (0.19 ; 0.70)	-0.03 (-0.16 ; 0.10)	7.7
	Weight gain from birth to two years	-0.02 (-0.40 ; 0.35)	-0.06 (-0.27 ; 0.14)	**71.7**
Android to gynoid fat ratio	Birth weight	0.014 (0.006 ; 0.022)	-0.002 (-0.006 ; 0.002)	17.1
	Weight gain from birth to two years	0.018 (0.005 ; 0.030)	0.001 (-0.006 ; 0.007)	3.4
Waist circumference (cm)	Birth weight	2.02 (1.28 ; 2.76)	0.18 (-0.22 ; 0.57)	8.1
	Weight gain from birth to two years	0.12 (-1.03 ; 1.26)	0.72 (0.09 ; 1.34)	**86.1**
Waist to height ratio	Birth weight	0.012 (0.007 ; 0.016)	0.001 (-0.002 ; 0.003)	3.0
	Weight gain from birth to two years	0.002 (-0.005 ; 0.009)	0.003 (-0.001 ; 0.007)	56.0
Lean mass index (kg/m^2)	Birth weight	0.37 (0.25 ; 0.48)	0.04 (-0.02 ; 0.10)	8.9
	Weight gain from birth to two years	0.16 (-0.02 ; 0.34)	-0.02 (-0.11 ; 0.08)	12.7
Height (cm)	Birth weight	-0.40 (-0.78 ; -0.02)	-0.18 (-0.39 ; 0.03)	30.9
	Weight gain from birth to two years	-0.93 (-1.52 ; -0.33)	0.10 (-0.24 ; 0.43)	11.5

For peer review only

Supplementary table 1. Description of follow-ups in the 1982 and 1993 cohorts, Pelotas - RS, Brazil.

1982 Cohort			1993 Cohort		
**Follow-up**	**Sample evaluated**	**Follow-up rate (%)**	**Follow-up**	**Sample evaluated**	**Follow-up rate (%)**
1982 (perinatal study)	All newborns	-	1993 (perinatal study)	All newborns	-
1983 (at 1st year)	All subjects born between January and April (1/3 of the cohort)	79,3	1993 (at 1st month)	Systematic sampling of 13% of cohort members	99,1
1984 (at 2 years)	All subjects	87,2	1993 (at 3 months)	Systematic sampling of 13% of cohort members	98,3
1986 (at 4 years)	All subjects	84,1	1993 (at 6 months)	All subjects with low birth weight and 20% of remnants	96,8
1997 (at 15 years)	All subjects residing in 27% of the city's census tracts	71,8	1994 (at 1st year)	All subjects with low birth weight and 20% of remnants	93,4
2000 (at 18 years)	All men	78,9	1997 (at 4 years)	All subjects with low birth weight and 20% of remnants	87,2
2001 (at 19 years)	All subjects residing in 27% of the city's census tracts	69,0	2004 (at 11 years)	All subjects	87,5
2004-2005 (at 23 years)	All subjects	77,4	2008 (at 15 years)	All subjects	85,7
2012-2013 (at 30 years)	All subjects	68,1	2011 (at 18 years)	All subjects	81,4
			2015-2016 (at 23 years)	All subjects	76,3

For peer review only

Supplementary table 2. Follow-up rate at 30 years (1982 cohort) and at 22 years of age (1993 cohort) according to baseline characteristics of the cohort.

Variable	1982 Cohort		1993 Cohort	
	Original cohort (n)	Followed at 30 years (%)	Original cohort (n)	Followed at 22 years (%)
**Sex**				
Male	3,037	65.2	2,603	72.6
Female	2,876	71.1	2,645	79.9
**Birthweight (g)**				
< 2500	534	72.1	510	79.2
2500 to 2999	1,393	69.1	1,409	75.7
3000 to 3499	2,220	66.0	2,030	75.9
≥ 3500	1,762	68.6	1,283	76.0
**Gestational age (weeks)**				
< 37	294	74.5	589	74.1
≥ 37	4,380	68.1	4,582	76.5
**Family income at birth (minimum wages)**				
< 1	1,288	66.1	967	75.1
1.1 to 3	2,789	70.4	2,260	74.8
3.1 to 6	1,091	69.3	1,204	79.6
6.1 to 10	382	61.3	433	75.7
≥ 10	335	60.3	385	74.6

For peer review only

Supplementary table 3. Maternal smoking during pregnancy according to baseline socioeconomic characteristics of the 1982 and 1993 cohorts.

	Duration of maternal smoking			
	**Non-smokers N (%)**	**Smoked in part of pregnancy N (%)**	**Smoked in whole Pregnancy N (%)**	**P-value**
**1982 Cohort**				
**Familiar income (tertile)**				< 0.0001
1st	682 (58.9)	90 (7.8)	385 (33.3)	
2nd	837 (63.9)	98 (7.5)	374 (28.6)	
3rd	880 (71.6)	93 (7.6)	255 (20.8)	
**Maternal schooling (years)**				< 0.0001
0 to 4	1,734 (62.4)	214 (7.7)	829 (29.9)	
5 to 8	273 (67.6)	33 (8.2)	98 (24.2)	
≥ 9	391 (76.5)	34 (6.7)	86 (16.8)	
**Partner smoking at 4 years**				0.562
No	771 (65.0)	82 (6.9)	334 (28.1)	
Yes	1,099 (65.0)	134 (7.9)	461 (27.2)	
**1993 Cohort**				
**Familiar income (tertile)**				< 0.0001
1st	978 (62.7)	67 (4.3)	514 (33.0)	
2nd	727 (66.4)	49 (4.5)	319 (29.1)	
3rd	813 (74.9)	49 (4.5)	224 (20.6)	
**Maternal schooling (years)**				< 0.0001
0 to 4	576 (57.1)	52 (5.1)	381 (37.8)	
5 to 8	1,185 (66.4)	86 (4.8)	513 (28.8)	
≥ 9	802 (79.2)	31 (3.1)	179 (17.7)	
**Partner smoking in pregnancy**				< 0.0001
No	1,318 (77.9)	100 (5.9)	274 (16.2)	
Yes	1,396 (58.0)	226 (9.4)	785 (32.6)	

For peer review only

For peer review only

STROBE 2007 (v4) Statement—Checklist of items that should be included in reports of *cohort studies*

Section/Topic	Item #	Recommendation	Reported on page #
**Title and abstract**	1	(*a*) Indicate the study's design with a commonly used term in the title or the abstract	1
		(*b*) Provide in the abstract an informative and balanced summary of what was done and what was found	2
**Introduction**			
Background/rationale	2	Explain the scientific background and rationale for the investigation being reported	4
Objectives	3	State specific objectives, including any prespecified hypotheses	5
**Methods**			
Study design	4	Present key elements of study design early in the paper	5
Setting	5	Describe the setting, locations, and relevant dates, including periods of recruitment, exposure, follow-up, and data collection	5 to 8
Participants	6	(*a*) Give the eligibility criteria, and the sources and methods of selection of participants. Describe methods of follow-up	5 to 8
		(*b*) For matched studies, give matching criteria and number of exposed and unexposed	Not applicable
Variables	7	Clearly define all outcomes, exposures, predictors, potential confounders, and effect modifiers. Give diagnostic criteria, if applicable	5 to 7
Data sources/ measurement	8*	For each variable of interest, give sources of data and details of methods of assessment (measurement). Describe comparability of assessment methods if there is more than one group	5 to 7
Bias	9	Describe any efforts to address potential sources of bias	5 to 7
Study size	10	Explain how the study size was arrived at	5 to 7
Quantitative variables	11	Explain how quantitative variables were handled in the analyses. If applicable, describe which groupings were chosen and why	5 to 7
Statistical methods	12	(*a*) Describe all statistical methods, including those used to control for confounding	7
		(*b*) Describe any methods used to examine subgroups and interactions	Not applicable
		(*c*) Explain how missing data were addressed	8
		(*d*) If applicable, explain how loss to follow-up was addressed	8
		(*e*) Describe any sensitivity analyses	Not applicable
**Results**			

For peer review only

Participants	13*	(a) Report numbers of individuals at each stage of study—eg numbers potentially eligible, examined for eligibility, confirmed eligible, included in the study, completing follow-up, and analysed	8
		(b) Give reasons for non-participation at each stage	8
		(c) Consider use of a flow diagram	8
Descriptive data	14*	(a) Give characteristics of study participants (eg demographic, clinical, social) and information on exposures and potential confounders	8 and 20
		(b) Indicate number of participants with missing data for each variable of interest	8 and 20
		(c) Summarise follow-up time (eg, average and total amount)	8 and 20
Outcome data	15*	Report numbers of outcome events or summary measures over time	8 and 20
Main results	16	(*a*) Give unadjusted estimates and, if applicable, confounder-adjusted estimates and their precision (eg, 95% confidence interval). Make clear which confounders were adjusted for and why they were included	8, 9 and 21 to 23
		(*b*) Report category boundaries when continuous variables were categorized	20
		(*c*) If relevant, consider translating estimates of relative risk into absolute risk for a meaningful time period	Not applicable
Other analyses	17	Report other analyses done—eg analyses of subgroups and interactions, and sensitivity analyses	Not applicable
**Discussion**			
Key results	18	Summarise key results with reference to study objectives	10
**Limitations**			
Interpretation	20	Give a cautious overall interpretation of results considering objectives, limitations, multiplicity of analyses, results from similar studies, and other relevant evidence	12
Generalisability	21	Discuss the generalisability (external validity) of the study results	12
**Other information**			
Funding	22	Give the source of funding and the role of the funders for the present study and, if applicable, for the original study on which the present article is based	13

*Give information separately for cases and controls in case-control studies and, if applicable, for exposed and unexposed groups in cohort and cross-sectional studies.

Note: An Explanation and Elaboration article discusses each checklist item and gives methodological background and published examples of transparent reporting. The STROBE checklist is best used in conjunction with this article (freely available on the Web sites of PLoS Medicine at http://www.plosmedicine.org/, Annals of Internal Medicine at http://www.annals.org/, and Epidemiology at http://www.epidem.com/). Information on the STROBE Initiative is available at www.strobe-statement.org.

For peer review only
